# Provider, sponsor and family perceptions of Child and Adult Care Food Program (CACFP) participation and COVID-19 reimbursement increases

**DOI:** 10.1017/S1368980025101389

**Published:** 2025-11-03

**Authors:** Kassandra A. Bacon, Danielle L. Lee, Reka Vasicsek, Celeste Felix, Samantha Kay-Daleiden Marshall, Elyse Homel Vitale, Susana L. Matias, Lorrene D. Ritchie

**Affiliations:** 1 Nutrition Policy Institute, University of California, Agriculture and Natural Resources, 1111 Franklin Street, Eleventh Floor, Oakland, CA 94607, USA; 2 CACFP Roundtable, PO Box 721002, San Diego, CA 92172, USA; 3 Department of Nutritional Sciences and Toxicology, University of California, Berkeley, 225 Morgan Hall, Berkeley, CA 94720, USA

**Keywords:** Nutrition policy, Child and adult care food program, Childcare, Reimbursement, Preschool-age children

## Abstract

**Objective:**

Declining participation by family childcare home (FCCH) providers in the Child and Adult Care Food Program (CACFP) may stem from inadequate tiered reimbursements for nutritious foods. During the COVID-19 pandemic, federal waivers temporarily eliminated tiers and increased reimbursements. We evaluated provider, sponsor and family perceptions of CACFP benefits and challenges in general and regarding the temporary removal of tiers and increased reimbursement rates.

**Design:**

From September 2023 to February 2024, FCCH providers, CACFP sponsors and CACFP family recipients in California participated in semi-structured interviews about CACFP benefits and challenges, perception of tiers and the COVID-19 waiver, quality of food and business viability. Thematic analysis was conducted using the immersion crystallisation method.

**Setting:**

Virtual interviews with California providers, sponsors and families.

**Participants:**

FCCH providers (*n* 31), CACFP sponsors (*n* 10) and CACFP family recipients (*n* 6).

**Results:**

Providers and sponsors reported that the higher temporary reimbursement rate positively impacted food budgets and quality. Pandemic-era facilitators of CACFP participation included the higher reimbursement rate, tier removal and a hybrid model for monitoring visits. Benefits beyond the pandemic included nutrition education and supporting child food security. Families valued CACFP for providing a variety of high-quality foods. However, barriers to CACFP participation persist, including administrative burden, inadequate reimbursements, strict regulations and the impacts of the pandemic and inflation.

**Conclusions:**

Increasing CACFP reimbursements while reducing other participation barriers can better support FCCH providers’ and sponsors’ participation. Supporting FCCH CACFP participation and retention can enhance access to healthy and nutritious meals for children from families with low income.

The Child and Adult Care Food Program (CACFP), funded by the United States Department of Agriculture, reimburses childcare centres and family childcare homes (FCCH) for serving nutritious foods and beverages to over 4·5 million children^(1,2)^. Children in FCCH are more often from families with low incomes than those in centres, as FCCH offer more affordable and flexible childcare^(3,4)^. Many young children consume up to two-thirds of their daily caloric intake at childcare, making CACFP integral to supporting low-income children’s access to nutritious foods during critical developmental years^(5–7)^.

CACFP reimburses childcare providers for up to two meals and one snack per child daily. Unlike centres, FCCH receives tiered reimbursements determined by provider income or geographical area, and they must work with a sponsoring organisation for programmatic administration^(8,9)^. Tier 1 reimbursements apply to FCCH in low-income areas or operated by a provider with a household income ≤ 185 % of the federal poverty level; otherwise, tier 2 rates apply^(8,10)^. Despite having similar nutritional and administrative requirements, tier 2 rates are almost half of tier 1 rates. While tiered CACFP rates, begun in 1997, aimed to support socio-economically disadvantaged families, this bifurcation contributed to a decline in CACFP participation^(10–13)^. A steady decline in CACFP sponsors also impacts CACFP access, exacerbated by the COVID-19 pandemic prompting many FCCH providers to close temporarily, widening disparities in access to healthy food and food security for young children^(3,14–16)^.

To address pandemic-related challenges, CACFP tiering was temporarily removed and reimbursements increased for all licensed FCCH from July 2021 to June 2023^(17,18)^. The maximum reimbursement increased from $4·78 for tier 1 and $2·29 for tier 2 to $5·67 for both tiers for claiming breakfast, lunch and a snack per child per day^(19)^. Additional waivers granted temporary programme implementation and oversight flexibilities, such as virtual monitoring to replace in-person monitoring by sponsoring organisations^(18)^. Our study aimed to understand FCCH providers’, sponsors’ and families’ perceptions of CACFP benefits and challenges in general and of the increased reimbursements and presumed challenges of reinstating tiers. Substantiated findings from semi-structured qualitative interviews can be utilised by federal and state CACFP agencies to enhance CACFP access and effectiveness.

## Methods

### Sample selection, recruitment and data collection

Three master’s degree-level female researchers trained in public health qualitative research methods (KB, DL and RV) conducted semi-structured phone and Zoom (V6.0.11, Zoom Video Communications Inc., 2023) interviews in English and Spanish with licensed FCCH providers (*n* 31), sponsors (*n* 10) and families with children in CACFP-participating FCCH (*n* 6) from California in September 2023–February 2024. In the spring of 2023, 2000 licensed California FCCH providers who participated in the CACFP were randomly selected from approximately 28 000 FCCH in state administrative data (obtained from the California Department of Social Services’ (CDDS) administrative CACFP data). Providers (*n* 518) completed an online survey as part of a mixed-methods study assessing the potential impacts of COVID-19 waivers, indicating interest in participating in an interview (*n* 256)^(20)^. The survey captured provider characteristics, including household food security using the six-item United States Department of Agriculture food security module^(21)^. To capture diverse perspectives, purposive sampling was used to select interview participants based on CACFP tier, years in business, language spoken, location (rural/urban), perceived impacts of tiered and untiered reimbursements and CACFP satisfaction^(22)^. A sample of seventy-five providers was selected for recruitment out of the 256 providers who opted into interview participation. All providers were invited via email and/or phone to participate in an interview; three refused, two were ineligible and thirty-nine were not further contacted after saturation was achieved with thirty-one interviews. Sponsors (*n* 10) were matched to interviewed providers, and all were interviewed to achieve saturation. Family participants were recruited from surveyed providers. Family interview saturation was achieved after six interviews. Interview guides (see online Supplemental Material) included open-ended questions developed by the research team and a study advisory board comprised of research, policy and community-based CACFP experts. Provider and sponsor topics included CACFP benefits and challenges, perceptions of reimbursements, business viability, quality of FCCH meals/snacks and food security. Family topics included the importance of childcare offering meals/snacks, CACFP awareness and childcare costs. Interview guides were pilot-tested with providers, families and sponsors on the advisory board.

Participants were recruited via email, phone and flyers and had no prior relationship to researchers. They provided oral consent and received a $50 gift card for participating in an interview. Interviews lasted between 6 and 63 min, averaging 25 min across the interview sample. Researchers documented field notes, and interviews were audio-recorded, transcribed and translated if not conducted in English; vocal disfluencies and personally identifiable data were removed. Transcripts and resulting coding were not returned to participants for comment.

### Data analysis

Immersion-crystallisation methodology informed transcript analysis, involving a hybrid approach of inductive and deductive coding^(23)^. Deductive coding allowed codes to be guided by the interview questions, and inductive codes were applied to themes naturally emerging. Three researchers reviewed transcripts (divided evenly) to identify initial themes and then discussed and revised the coding scheme to develop an initial codebook. This reciprocal process continued until a final coding scheme (see online Supplemental Material) was developed, approved by the principal investigator (LDR) and uploaded to Atlas.ti (Scientific Software Development GmbH. V23·2, 2023). The three researchers were randomly assigned a subset of transcripts to code. Transcripts were reviewed by a second coder, differences were discussed and triangulation was used to increase validity and reliability^(24)^.

## Results

### Participant characteristics

FCCH (*n* 15 tier 1, *n* 16 tier 2) socio-demographic characteristics are shown in Table 1. Providers were licensed for an average of 13 years, with nearly two-thirds licensed to care for up to 14 children. On average, each FCCH employed two staff members, including the provider, and took care of ten children, most over one year old. English was the primary language of most children. All providers offered full-day care, and some also provided half-day care; few offered weekend, evening or overnight care. Tier 1 eligibility was mostly determined using school-level area eligibility data. Two meals and two snacks were most commonly provided and claimed for CACFP reimbursement, with lunch, breakfast and mid-morning or mid-afternoon snacks being the most common. Most providers participated in CACFP for over 10 years. Providers were on average 51 years old, all female and half were Hispanic or Latino(a) with some college education or an Associate’s degree. A majority (86 %) had incomes at or below the federal poverty level, and 31 % experienced household food insecurity (those who were deemed to have ‘Low’ or ‘Very Low’ food security). Sponsors (*n* 10) represented eight of forty-one organisations across twenty-four of fifty-eight California counties. Two were sponsors for FCCH providers interviewed; eight were sponsors of FCCH providers surveyed but not interviewed. Over 60 % of sponsoring organisations provided multilingual services in Spanish (all), Mandarin or simplified Chinese (half) and one sponsor in Tagalog, Russian and Ukrainian. One-third of families interviewed were Spanish-speaking. Two were from interviewed FCCH providers; five were from surveyed FCCH providers. The family sample was evenly split between those with 1 or 2 children attending the FCCH and between those attending for more or less than 12 months. Only one individual, the primary caregiver, from each family was interviewed.

**Table 1.** Characteristics of licensed family childcare home providers that participate in the child and adult care food program (CACFP) who completed interviews (*n* 31)

Characteristic^*^	*n*	%
Family childcare homes		
No. of years licensed		
Mean	13·2	
sd	9·2	
Facility capacity		
Up to 8 children (small)	12·0	38·7
Up to 14 children (large)	19·0	61·3
CACFP tier 1 provider	15·0	48·4
Tier eligibility determination		
Income	1·0	6·7
Area eligibility	14·0	93·4
	Mean	sd
No. of staff (mean, sd)	2·5	1·4
Total children in care, by age (mean, sd)	10·4	7·1
0–11 months	0·7	1·5
12–23 months	1·6	1·2
24–35 months	2·8	2·8
3–5 years	3·7	3·1
6+ years	1·7	3·3
	*n*	%
Family primary language†		
English	29·0	93·5
Spanish	5·0	16·1
Chinese	2·0	6·5
Other	3·0	9·7
Type of care provided^†^		
Full day	31·0	100·0
Half day	12·0	38·7
Weekends	2·0	6·5
Evenings	3·0	9·7
Overnight	1·0	3·2
Meals/snacks provided		
Breakfast	27·0	87·1
Lunch	31·0	100·0
Dinner	15·0	48·4
Mid-morning snack	22·0	73·3
Mid-afternoon snack	29·0	96·7
Evening snack	10·0	32·3
Meals/snacks claimed from CACFP		
Breakfast	23·0	74·2
Lunch	31·0	100·0
Dinner/Supper	11·0	35·5
Mid-morning snack	18·0	58·1
Mid-afternoon snack	24·0	77·4
Evening snack	4·0	12·9
Years in CACFP		
1–3 years	4·0	12·9
3–5 years	2·0	6·5
5–10 years	6·0	19·4
10+ years	19·0	61·3
Family child care home provider		
Age		
Mean	51·2	
sd	10·5	
Female	31·0	100·0
Race/Ethnicity^†^,^‡^		
Non-Hispanic Asian/Pacific Islander	4·0	12·9
Non-Hispanic Black/African American	2·0	6·5
Hispanic or Latino(a)	17·0	54·8
Non-Hispanic White	5·0	16·1
Mixed	2·0	6·5
Other	1·0	3·2
Education level		
HS graduate	3·0	9·7
Some college or Associate’s degree	17·0	54·8
Bachelor’s degree	8·0	25·8
Master’s degree or higher	3·0	9·7
Household income before taxes^§^		
< 100 % FPL	5·0	17·9
100 % FPL	19·0	67·9
200 % FPL	4·0	14·3
Household food security||		
High	26·0	83·9
Low	3·0	9·7
Very low	2·0	6·5

CACFP, Child and Adult Care Food Program; FPL, federal poverty level.

* Frequency and percent are presented unless otherwise specified.

† Percent may add up to >100 % as participants could select more than one answer option.

‡ Answer options included ‘Non-Hispanic Native American/American Indian’. No participant selected this answer option.

§ Determined based on the Federal US Department of Health and Human Services Poverty Guidelines for 2023, which are used to determine financial eligibility for certain programs^(38)^.

|| Assessed using the USDA six-item food security module^(21)^.

#### Interview themes and subthemes

**Figure 1.**
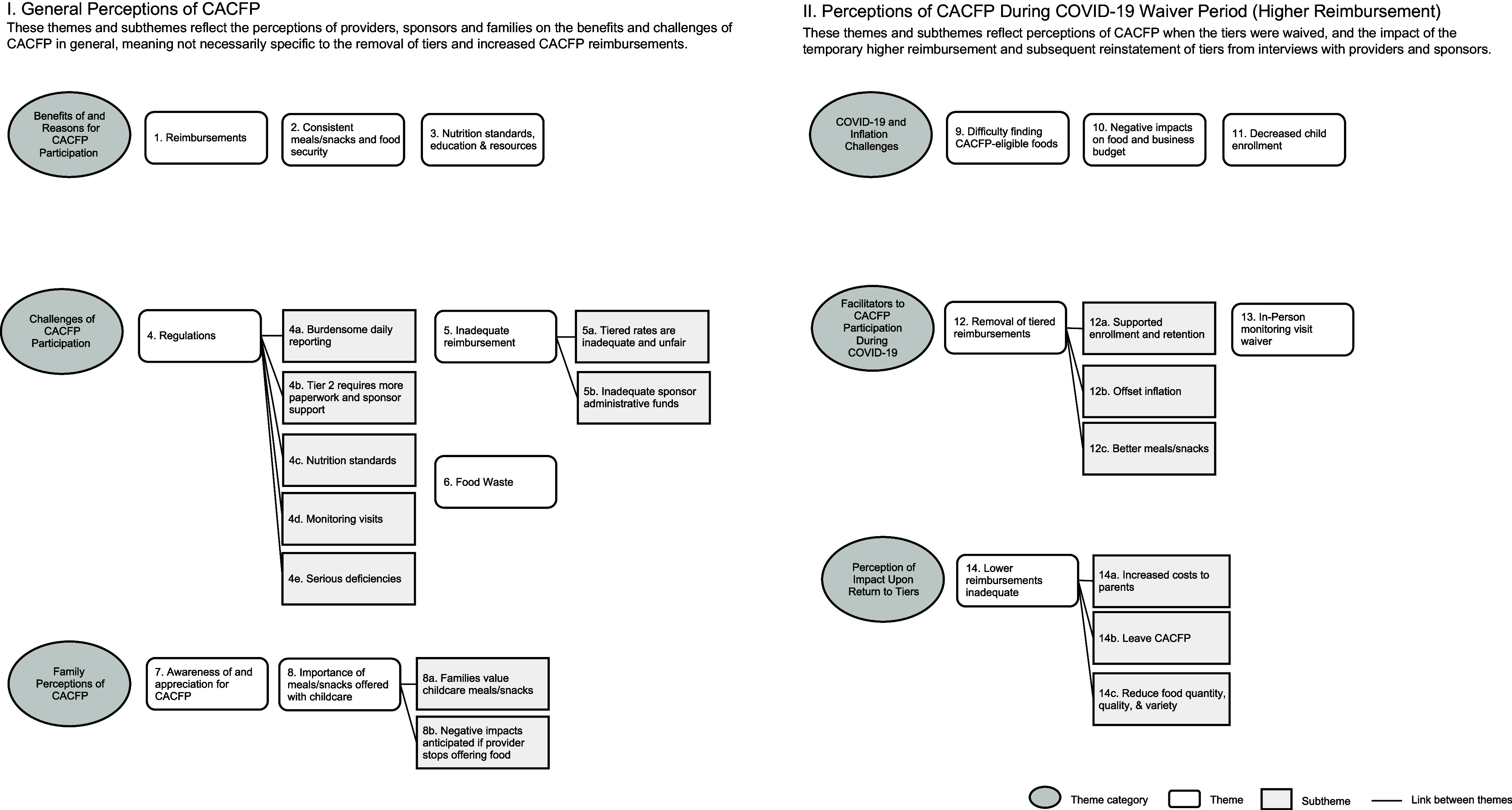
Themes and sub-themes identified during qualitative interviews with licensed family childcare home providers that participate in the Child and Adult Care Food Program (CACFP) (*n* 31), CACFP sponsors (*n* 10) and CACFP family recipients (*n* 6) in California.

Themes and subthemes from interviews with CACFP providers, sponsors and families are reported in Figure 1 and see online Supplemental Table A.

##### I. General Perceptions of CACFP

These themes and subthemes reflect the perceptions of providers, sponsors and families on the benefits and challenges of CACFP in general, meaning they are not necessarily specific to the removal of tiers and increased CACFP reimbursements.

Benefits of and Reasons for CACFP Participation

*Theme 1. Reimbursements.* Providers and sponsors cited meal/snack reimbursements as a primary reason for, benefit and facilitator of CACFP participation. Reimbursements offset food costs and facilitated the purchase of ‘expensive’ higher-quality food that adhered to CACFP standards: whole grains, fresh fruits and vegetables and lean proteins.

*‘The food program, they help us the most with the high cost of the healthy food, it’s expensive.’* Tier 1

*Theme 2. Consistent meals/snacks and food security.* Providers and sponsors said CACFP supports providers in offering consistent and healthy meals/snacks, particularly for families experiencing food insecurity. Several providers noted that children arrive hungry, and they worry children experience hunger over the weekends. They also noted that CACFP foods were more nutritious and consistent than what children receive at home.

*‘I know for some of the kids, they are the only real meals they eat… when they go home to dinner a lot of times it’s just a sandwich or cereal.’* Tier 2

*Theme 3. Nutrition standards, education and resources.* Providers and sponsors said CACFP increased their awareness about what and how to feed children through nutrition standards, education and resources. Providers appreciated the trainings and resources available from CACFP.

*‘I also appreciate the trainings where we know how to identify what food is healthy. I have had my ‘aha’ moments when I thought it was getting something healthy and then it turns out, it had a lot of sugar, or it didn’t really have fiber.’* Tier 2

Challenges of CACFP Participation

*Theme 4. Regulations.* Providers and sponsors cited CACFP regulations as a major challenge to provider participation.

*Theme 4a. Burdensome daily reporting*. Daily reporting was the most common challenge for providers, saying it was overly laborious, difficult with their busy schedules and stressful. Providers requested weekly instead of daily reporting, expressing frustration for lost meals/snack reimbursement claims if they failed to submit on a given day.

*‘Us asking them to do all that paperwork sometimes they just feel like that’s not worth it for the money.’* Sponsor

*Theme 4b. Tier 2 requires more paperwork and sponsor support.* Tier 2 providers experienced difficulty having income-eligible families complete meal benefit forms (which can be used to attain higher reimbursements) due to families’ discomfort in reporting their income. Sponsors said tier 2 determination to ensure maximal reimbursement was administratively burdensome. Sponsors indicated fewer tier 2 than tier 1 providers participated in CACFP due to the burdensome daily reporting and tiering determination outweighing the tier 2 reimbursement rates.

*‘[Tier 2 providers] don’t feel comfortable asking their clients about their income … it’s just a touchy subject.’* Sponsor

*Theme 4c. Nutrition standards.* While providers appreciated that the CACFP nutrition standards resulted in health benefits for children, some providers found adhering to the meal patterns and nutrition standards challenging, citing difficulty finding CACFP-eligible foods. Sponsors indicated that program requirements can be ‘rigid’, and misunderstandings of CACFP nutrition standards can result in meal/snack reimbursement disallowances.

*‘They’re having trouble finding the 1 % milk or sometimes they still get confused about that whole grain-rich item rule. Because maybe the label says it’s made with whole grain, but that’s different from 100 %, or at least 50 % whole grains. So those are the types of barriers that some of our providers are facing.’* Sponsor

*Theme 4d. Monitoring visits.* Providers and sponsors reported in-person monitoring visits were challenging, time-consuming, disrupted providers’ daily routine and resulted in sponsor-related travel challenges, including stress due to traffic, adverse weather or when providers/children were not onsite or not serving a meal/snack when they arrived. Sponsors said travel was expensive, parking was difficult and expressed safety concerns at some visits.

*‘The main challenge is accommodating the inspection… accommodating the visitor and talking to her and giving the information, it’s a lot of additional work that we are doing, especially when it is in person. If it is virtual, then it was very easy for us the last two years.’* Tier 2

*Theme 4e. Serious deficiencies.* Sponsors said the serious deficiency process (which occurs when providers do not adhere to CACFP regulations) hindered program participation. Sponsors reported this process was ‘too harsh’ and ‘time-consuming’. Sponsors perceived inequities in the process, especially when compliance issues stemmed from language barriers faced by non-English-speaking providers.

*‘Some of the regulations are a little too harsh as far as if you’re found to be non-compliant and you’re put in the serious deficiency process. The seven years of being on the national disqualified list… it becomes a hindrance to them [providers], just knowing the possibilities.’* Sponsor

*Theme 5. Inadequate reimbursement.* Inadequate meal/snack reimbursement was another major challenge cited by sponsors and providers (pre-, during- and post-pandemic).

*Theme 5a. Tiered rates are inadequate and unfair.* Although providers expressed gratitude for the reimbursements, providers and sponsors perceived the tiered rates as inadequate to cover food costs and not worth the effort required to participate in CACFP. Sponsors and providers said tiered reimbursements were unfair, inequitable and administratively burdensome. Most advocated for universal tier 1 rates to improve equity.

*‘The funding is a big challenge… I’m charging everybody [families] the same, but it’s very difficult to feed a child a healthy fresh fruit, whole grain for 25 cents per child. You just can’t do it… A lot of people are choosing to quit the program because they say it’s not worth it.’* Tier 1

*Theme 5b. Inadequate sponsor administrative funds.* A challenge cited by sponsors was the lack of staff funding to support providers effectively. This dovetailed with decreased childcare enrollment and decreased CACFP reimbursement claims which contributed to inadequate funding for staff and FCCH resources.

*‘The admin rate isn’t sufficient to cover the cost to run the program… I don’t think that this program is designed to have full time workers with benefits. It’s just not set up with enough funds coming to admins [administrators] for that.’* Sponsor

*Theme 6. Food waste.* Providers and sponsors cited difficulty getting the children to consume CACFP meals/snacks, particularly milk and vegetables, resulting in provider frustration. They perceived this as a ‘waste of food’ and a ‘waste of money’.

*‘Following all the rules that I have to follow, as far as the amount. Sometimes the kids eat, sometimes they don’t. I waste a lot of food at times.’* Tier 2

Family Perceptions of CACFP

*Theme 7. Awareness of and appreciation for CACFP.* Those families aware of CACFP cited knowing it enabled providers to serve nutritious meals/snacks that providers were reimbursed for. When informed by the interviewer that their FCCH provider was only partially reimbursed for meals/snacks, one family member said knowing this made them ‘sad’ and another desired that providers be fully reimbursed, suggesting families cover the difference or that the additional cost be reflected in their childcare rates. Those aware of CACFP expressed appreciation for being a part of the program.

*‘We are a big fan of it [CACFP]. It was not something we were familiar with before starting with our provider… it’s a really nice perk. We like it. We like participating in it.’* Family

*Theme 8. Importance of meals/snacks offered with childcare.*

Families cited the meals/snacks offered as part of their childcare was important to them.

*Theme 8a. Families value childcare meals/snacks.* All families interviewed found it helpful that their FCCH provided meals/snacks. Most mentioned that this influenced their childcare choice, ensuring their children received nutritious options while saving the family time and money. Families overwhelmingly reported that the quality and variety of meals/snacks served were important. When probed to expand on why, responses included a desire to have their children consume ‘natural’, ‘less processed’ and ‘fresh food’. Variety was cited as a way to expand a child’s palate and offer healthy foods that they may not have access to at home. Providers and sponsors also stated that CACFP improves parental perception of the quality of the meals/snacks served by their FCCH.

*‘I think it’s definitely important that children try any and all foods, especially vegetables… they may be trying things that I don’t serve at home.’* Family

*Theme 8b. Negative impacts anticipated if provider stops offering food.* Families reported varying perceived negative impacts if their provider stopped offering meals/snacks, including increased food costs and challenges related to time required to manage meal purchasing, preparation and packing. Several said this may diminish the variety and nutrition of children’s meals/snacks, citing the meals/snacks they prepare are less nutritious than what their provider offers, and may result in them changing providers.

*‘It would definitely add stress to my plate…I know she’s [daughter] getting the variety and the nutrition [in child care meals]. So I feel that would maybe diminish if I was having to pack her lunch each day. And then also financially, I know that I would be buying more food if I was providing her meals.’* Family

##### II. Perceptions of CACFP During COVID-19 Waiver Period (Higher Reimbursement)

These themes and subthemes reflect perceptions of CACFP when the tiers were waived and the impact of the temporary higher reimbursement and subsequent reinstatement of tiers from interviews with providers and sponsors.

COVID-19 and Inflation Challenges

Many providers and sponsors noted several challenges to operating CACFP during the COVID-19 pandemic. When asked about perceptions of the temporary higher reimbursement and reinstatement of tiers, providers and sponsors had a difficult time separating the perceived impacts from the concurrent impacts of the pandemic amid ongoing inflation affecting their business costs.

*Theme 9. Difficulty finding CACFP-eligible foods.* Providers and sponsors cited challenges finding CACFP-eligible foods due to supply chain issues during the COVID-19 pandemic. Providers and sponsors indicated that certain items, milk especially, were difficult to obtain or completely unavailable. Providers reported more frequent grocery store visits to find competitive prices for CACFP-eligible items.

*‘Being able to get certain items when the pandemic first happened, there was an issue with getting milk, certain items, the stores and shelves were bare.’* Sponsor

*Theme 10. Negative impacts on food and business budget.* Providers reported negative effects of economic instability due to COVID-19 and inflation on their business budgets, resulting in some increasing their childcare rates and reducing other business costs, such as for curriculum and materials. Inflation led some providers to pay out of their personal budget for food. Some sponsors observed that providers chose cheaper, frozen or packaged items to deal with budget constraints due to COVID-19 and inflation.

*‘The biggest challenge… right now [is] inflation, that food [cost] has gone up a lot… to be able to provide good food to the children and that we can afford…’* Tier 1

*Theme 11. Decreased child enrollment.* Providers and sponsors indicated that enrollment was particularly impacted by the pandemic, leading to reduced CACFP claims and thus less reimbursement which demotivated FCCH program participation and reduced sponsor administrative funds.

*‘During the pandemic we lost lots of our kids. And we lost the food program money, because we don’t have the kids.’* Tier 1

*‘I think because of the pandemic we were losing providers because they didn’t have children, or they didn’t want to stay offering childcare because they were older, and they didn’t want to expose themselves to COVID. So, we lost a lot of providers on that end and that affected our claiming, v. how much they were getting paid.’* Sponsor

Facilitators of CACFP Participation During COVID-19

*Theme 12. Removal of tiered reimbursements.* Providers, particularly tier 2, and sponsors cited the removal of tiering and resulting higher reimbursement during the pandemic as a facilitator to CACFP participation.

*Theme 12a. Supported enrollment and retention.* The higher reimbursement encouraged new providers to join the program. Providers and sponsors noted the removal of tiering also encouraged program retention.

*‘The higher COVID rate is most sufficient for me to stay on the food program and be able to provide nutritious meals for the kids.’* Tier 2

*Theme 12b. Offset inflation.* Some providers said the higher reimbursement helped with purchasing foods as costs increased due to inflation. However, providers and sponsors still perceived the higher reimbursement as inadequate to cover food costs.

*‘[The higher reimbursement] evened out because of prices at the grocery store… some of them doubled. When the food program was paying a little higher, it literally leveled out because what they were paying is what I was paying out, so I’m almost breaking even from what they give me from what I buy.’* Tier 1

*Theme 12c. Better meals/snacks.* Some providers said the higher reimbursement improved quality and variety of meals/snacks offered, particularly for fruits and vegetables. They highlighted increased organic, seasonal and local foods, which they perceived to be of higher quality. The majority of providers reported not changing the number of meals/snacks offered despite the higher reimbursement, though some providers and sponsors said the higher reimbursement allowed providers to offer more food beyond CACFP-reimbursed meals/snacks, including sending children home with food who may not have access to enough food at home.

*‘I was able to provide more variety of fresh fruits and vegetables with the higher rate.’* Tier 2

*‘Definitely [when] you get paid more, you serve better. You can serve to those families in need. You make sure they eat and make sure you give the packs of food to go during the night, if he is hungry or he wants food, he has food.’* Tier 1

*Theme 13. In-person monitoring waiver.* The temporary COVID-19 waiver for in-person monitoring visits also facilitated CACFP participation. Sponsors could schedule online video meetings with providers to observe meal/snacks, which were a positive experience for providers and alleviated sponsors’ travel challenges. Most sponsors advocated for virtual-only monitoring visits. Others suggested a hybrid model, where two out of three required annual visits were virtual, reporting difficulties observing what and how much was being served and providing technical assistance when visits were only virtual.

*‘When we were doing the COVID protocol for visits [virtual], it was much easier to save time and also be able to maximize meal observations.’* Sponsor

Perception of Impact Upon Return to Tiers

*Theme 14. Lower reimbursements inadequate.* Providers and sponsors perceived that returning to tiered reimbursements would result or already had resulted in inadequate funding, especially due to inflation and for tier 2 providers.

*Theme 14a. Increase costs to parents.* Some providers anticipated passing along food costs to parents by increasing their childcare rates or serving fewer meals/snacks when tiers returned. However, many providers were not comfortable increasing their childcare rates despite lower CACFP reimbursements. To deal with reduced reimbursements, providers said they would continue offering meals/snacks but serve less or ask parents to contribute some foods; other might ask parents to provide all meals/snacks.

*‘We may end up having to drop the amount of food we provide and ask parents to bring in additional food. Or, if for example, the child is hungry after the amount of food that we’re providing, that parent might have to supplement with food.’* Tier 2

*Theme 14b. Leave CACFP.* Some providers, especially Tier 2, said they may leave CACFP when tiers were reinstated, citing it was not worth the effort to participate if they received less funding.

*‘It might affect my ability to participate [in CACFP] if I’m not being reimbursed enough to purchase groceries for my program.’* Tier 2

*Theme 14c. Reduce food quantity, quality and variety.* Providers said they would reduce food quality and variety and limit offering seconds or reduce portions. Several mentioned they may offer more canned and processed foods, which they cited as less costly than fresh, whole foods. Providers said they would need to spend more of their own money to meet CACFP standards and stay in the program.

*‘I am looking for ways to cut down the cost. That [reduced reimbursement] has a direct effect on my quality. I reduced the number of times I provide a snack.’* Tier 2

## Discussion

This California study illuminated several perceived benefits of and reasons for participating in CACFP, challenges of CACFP participation and family perceptions of CACFP in general. It also elucidated perceptions of CACFP during the COVID-19 pandemic when FCCH providers received temporary higher reimbursement rates, including pandemic- and inflation-related challenges, facilitators to CACFP participation during the pandemic and perception of the impact of return to tiering post-pandemic. A consistently reported CACFP benefit cited by providers, sponsors and families was funding to provide nutritious meals/snacks to young children. However, providers and sponsors perceived the reimbursements as inadequate (pre-, during and post-pandemic) to cover the full cost of food to meet CACFP meal standards. Additionally, sponsors reported difficulty re-instituting tiered rates because of the administrative burden, deeming tiering inequitable and a barrier to CACFP participation, especially for tier 2 providers. Most providers and sponsors advocated for an increased reimbursement rate for all providers. Given that reimbursement inadequacy have been substantiated as a key barrier to CACFP access in prior studies and that FCCH participation is steadily declining, our study findings reinforce the need for removal of the tiered reimbursements^(10–12,25–29)^.

Families reported valuing nutritious foods served by CACFP-participating FCCH, emphasizing the importance of exposing their children to a variety of foods to overcome picky eating and establish healthy eating patterns. Interviews with families, providers and sponsors illustrated that more could be done to improve awareness of CACFP to increase programme participation. Providers and sponsors reported challenges to CACFP participation, which included difficulty with CACFP nutrition standards, daily reporting of meals/snacks, the serious deficiency process and in-person monitoring visits. These challenges align with findings from other studies^(10–12,26,27,30)^. Providers perceived daily reporting as restrictive and onerous, and sponsors suggested the benefits of CACFP may not outweigh the administrative burden for all providers. The three annual in-person monitoring visits (two unannounced) were another barrier^(31)^. Providers felt the unannounced visits were disruptive, and sponsors had difficulty related to travel. Providers and sponsors reported positive experiences with the virtual monitoring allowed during the pandemic. While sponsors reported valuing in-person monitoring to better capture mealtime experiences, they advocated for a hybrid model to enhance the monitoring experience for providers and reduce travel costs for sponsors, which could enable use of administrative funds for other CACFP supports. These findings are consistent with another study, which found that meal pattern integrity may be compromised with virtual monitoring, but it granted staff more flexibility and time to support providers in other ways^(32)^.

This study illustrated facilitators to CACFP participation during the COVID-19 pandemic, specifically the elimination of tiering and higher COVID-19 reimbursement rates for all FCCH providers. Many providers felt the higher rates helped offset the high cost of food due to inflation. According to the U.S. Consumer Price Index, increases to food at home prices peaked at 11·4 % between 2021 and 2022 and decreased to 5 % between 2023 and 2024. However, over the past year (July 2023–2024), food at home prices have increased by 1·1 %^(33)^. Some providers also attributed improved quality and variety of foods served, specifically for fruits and vegetables, to the higher reimbursement rate. These findings are consistent with a pre-pandemic study conducted in Washington state, showing that higher reimbursement rates received by tier 1 compared to tier 2 are associated with higher nutritional quality of foods served in FCCH^(34)^. Our study findings highlight the need to eliminate tiering and increase overall reimbursement rates for FCCH providers, ensuring they have adequate funding to provide children with a variety of high-quality foods and beverages.

In July 2023, the temporary higher reimbursements expired, and tiering was reinstated. During the pandemic, all providers received $5·67 per child for claiming breakfast, lunch and a snack; current rates are $5·74 for tier 1 and $2·76 for tier 2^(35)^. Many providers and sponsors anticipated challenges with the return to tiering, specifically reduced quality and variety of foods, increased childcare fees, asking parents to provide meals/snacks and potentially leaving the program. Families also expressed concern over providers discontinuing childcare meals/snacks, citing time and financial burden and decreased nutritional quality of foods packed from home. This is particularly concerning given the nationwide decline in CACFP participation, which may reduce access to nutritious meals for children experiencing poverty and food insecurity^(10,36)^.

This study has several strengths. It is among the first qualitative studies to assess the impacts of the temporary higher reimbursement rate on providers, sponsors and families. While several studies have focused on providers and sponsors, few include family perceptions. Our qualitative study design provides a nuanced understanding of the perceived benefits and challenges of CACFP participation and the impacts of the higher reimbursement rate for providers and sponsors. This study was also strengthened by a relatively large sample size of providers, though research indicates qualitative saturation can be reached with smaller sample sizes^(37)^.

One limitation of this study is that findings may not be generalisable. Despite efforts to ensure the sampling method resulted in a diverse representation of CACFP-participating FCCH providers and sponsors across the state, self-selection bias is possible. Those who self-selected to complete the survey and participate in an interview may be systematically different from those who did not. Selection bias may also exist among families interviewed, as they were recruited by FCCH providers. Families included through provider selection may share characteristics that set them apart from others who were not included. Additionally, social desirability and confirmation bias may have influenced results, though study findings were substantiated across multiple interviews, and researcher triangulation was performed to reduce bias^(24)^. Perceived impacts of the higher reimbursement rate, albeit applied to providers nationally, may vary in other states due to CACFP administration, cost of living or other differences. Finally, though providers and sponsors were asked about their perceived impact of the return to tiers, some providers had already started receiving the lower reimbursement rate at the time of the interview, which may have impacted their responses. Subsequent studies should assess the actual impacts now that tiers have been reinstated.

## Conclusion

While providers and sponsors perceived positive impacts on the quality and variety of food served and food security among young children from the temporary elimination of tiering and higher COVID-19 reimbursement, barriers to CACFP remain as reimbursements may not outweigh the administrative costs associated with operating the program, especially during times of economic instability and inflation. CACFP policymakers should consider elimination of tiers and increasing the reimbursement rates to more adequately cover costs and allow virtual monitoring visits to support FCCH CACFP participation and retention. Finally, efforts could be made to improve awareness of the program among families, as this could be a potential driver of CACFP participation for FCCH providers.

## Supporting information

Bacon et al. supplementary material 1 Bacon et al. supplementary material

Bacon et al. supplementary material 2 Bacon et al. supplementary material

Bacon et al. supplementary material 3 Bacon et al. supplementary material

## Supplementary material

For supplementary material accompanying this paper visit https://doi.org/10.1017/S1368980025101389
