# Supplementary material for: Provider, sponsor and family perceptions of Child and Adult Care Food Program (CACFP) participation and COVID-19 reimbursement increases
Source: Public Health Nutr. 2025 Nov 3;28(1):e195. doi: 10.1017/S1368980025101389 (PMC12722094; doi:10.1017/S1368980025101389)
Supplement: Bacon et al. supplementary material 1 — Bacon et al. supplementary material [file S1368980025101389sup001.docx]

Supplemental Table A. Themes, Subthemes and Example Quotes from Child and Adult Care Food Program (CACFP) Family Childcare Home Providers, Sponsors and Family Recipients in California who Completed Interviews

| **I. General Perceptions of CACFP**  These themes and subthemes reflect the perceptions of providers, sponsors and families on the benefits and challenges of CACFP in general, meaning they are not necessarily specific to the removal of tiers and increased CACFP reimbursements. | | | | |
| --- | --- | --- | --- | --- |
| **Theme** | **Subtheme** | | **Description** | **Example Quote(s)** |
| **Benefits of and Reasons for CACFP Participation** |  | |  |  |
| 1. Reimbursements |  | | CACFP reimbursements offset food costs and help providers buy higher quality food. | “It's a great help for me to get some financial extra money, that I can hopefully not have to pass down to parents, because food costs especially after the pandemic has gone through the roof. It does really help for me to manage costs, operating costs, and then I can pass it on to a little bit higher salaries for my teachers, and hopefully a little lower prices for my parents.” Tier 2  “The very greatest benefit is getting reimbursed for the foods that I'm going to provide for the children regardless. That's the main factor.” Tier 2    “This helps knowing that I can go out and buy the food that I need to buy, fresh foods, high quality foods.” Tier 2    “It helps subsidize the meals so that I can feed the children a better quality of food.” Tier 2    “The food program, they help us the most with the high cost of the healthy food, it's expensive.” Tier 1    “Greatest benefit. I'd say being able to purchase food for the kids and make sure that they have healthy meals.” Tier 2    “We already serve, hot lunch, you know, and snacks to the families. And we do not charge additional for that cost. So this is a very nice way, you know, of bringing additional revenue for the business.” Tier 1  “It helped to reimburse my costs for the food that I fed the kids. I had six kids right off the bat. That was an expense for me, having to feed six kids that aren't my own kids. To have a little bit of reimbursement was helpful.” Tier 2    “The food program helps me when it comes to providing food. The funding that they give, I know it's not much but it's still something.” Tier 1    “When I started, it's like a bonus. I was starting my business, I needed income. I know that this will not cover the food completely, but at least something will help.” Tier 2    “It does help reimburse some of the monies that we—nowadays—spend quite a bit on the healthier foods.” Tier 1    “The cost reimbursement is really helpful.” Tier 2    “The benefits would be the amount that they give, which isn't as big of an amount that we get reimbursed.” Tier 1    “Well, financially, it's nice. It's not huge, because it's actually minimal now.” Tier 1    “We conducted a provider feedback survey and we found that the majority of our providers who participate in CACFP is because of the assistance with food costs.” Sponsor    “I know reimbursement for some of our providers is a top priority for them, especially right now with inflation being kind of high, food prices being high, they really want to see their dollar stretch and really want to see that their reimbursement, although they're understanding that it's not a full reimbursement, but at least it's really helping them.” Sponsor  “It's the financial aspect. I know that it's not a full reimbursement but just having that reimbursement come back to them to help meet the needs of the kids in their care and the program needs. It's a big help, it's not enough is what we always hear, but it is definitely something that helps them continue providing the meals and continue participating with the program.” Sponsor    “Well, obviously it's the cash reimbursement, with inflation and groceries and you know, even before inflation and groceries, food is probably the top thing on their [providers] list of expenses that they have to shell out to run their childcare.” Sponsor    “I know that a lot of providers have said that it does help keep the cost of overall daycare services down, which can then add benefits to families who live in the region that we cover.” Sponsor    “Some of the benefits are obviously the reimbursement. You know, it's one of the big ones that attracts most of our family child care homes, that they get monetary reimbursement.” Sponsor |
| 2. Consistent meals/snacks and food security |  | | Providing kids with consistent and healthy meals/snacks, supporting children who may experience food insecurity. | “The greatest benefit is just for me ensuring that I am serving nutritious meals every day.” Tier 1  "The best meals they eat are here with me... On the weekends, they're busy and running with their families and who knows what they get, probably one of those little squeeze packs of food, which I won't do. I think they get a lot of benefit from [the food program].” Tier 1  “I continue to make it easier for the parents to not worry in the morning what to feed their children. Plus, sometimes parents bring me junk, you know, they stop at McDonald's and grab a breakfast and bring it here and children spent ten-hours plus in my home, and I feel responsible to feed them good food, homemade food. Because the food like gas for their body, they need it, just like the gas for our cars, so I need to make sure that the children while under my care are well fed.” Tier 2  “We have picky eaters, and it gives them a good variety. And a lot of kids eat here more than they eat with their own families. So, I want to make it count.” Tier 1  “…making sure that all of the children get to have the same type of food so that way, it allows the children to eat at their own pace and get healthy meal and also help to quell that somebody brought a candy bar, and somebody brought Cheeze-its. Kids are going to want that over whatever they're having. With being on the food program, everybody gets the same food, so there isn't that type of stress of somebody has such-and-such and somebody else doesn't.” Tier 2  “I know for some of the kids, they are the only real meals they eat. I'd say over half of the children at my daycare. A full balanced breakfast and a full balanced lunch and balanced snack. I also have school aged children now, so when they go to school those same children eat the school lunch. And so, they are basically the only real meals the kids get me. When they go home to dinner a lot of times it's just a sandwich or cereal.” Tier 2  “I would say 85% of the children that are in daycare, receive their only nutritious meal in the daycare while they're in care versus what they receive at home.” Sponsor  “I know that for some of the kids of the providers that we sponsor, this is their only hot meal, or meals or snacks that they get on any given day.” Sponsor  “They're [providers] able to serve more nutritious meals. You know, food costs are so expensive right now, that being on the food program, I'm sure helps with that cost. That and the children receive well balanced, nutritious meals too at the same time.” Sponsor  “Children are receiving healthy, nutritious meals with a variety of fresh fruits and vegetables and lean meats. And fluid milk every day.” Sponsor  “Because if I would have to buy all the food for my daycare, I might not have as much to be able to help send that food to my families. To be able to help provide extra money to be able to do those things. The food program covers a lot of stuff in my daycare, and like I said, when I have extra money left over, I do things like that, to be able to make sure that nobody's going hungry.” Tier 1    “It was a really big hardship for providers to find or allocate foods when times were hard and with the food increases, I can assume that the parents are also dealing with that. I'm sure that it [higher reimbursement] did alleviate a lot of stress for families to know that they're in a home where they're being provided the meals that they need in a day… I do think it creates a sense of security for families.” Sponsor    “I had a provider in tears. She was a tier two provider, and she was in tears. When she heard that they were going to give them the higher rate and that they were all going to get the tier one, she was crying. She said “You have no idea how much of a difference this is for us. What I'm going to be able to provide the kids in my care, you know, you know, my own family.” Sponsor  “[CACFP] gives me a little extra to be able to help my other families that don't have the food on the table… I don't really give out money much, but I do make sure the food goes out.” Tier 1 |
| 3. Nutrition standards, education, and resources |  | | Increases providers’ awareness about what and how to feed children through nutrition standards, education and resources. | “It's been very valuable teaching me about how children eat and the different aspects of nutritional values of food.” Tier 2    “It's to help educate myself and the families on healthy eating habits for the kids.” Tier 1    “You also get... nutrition information from the program. So at least on a high level we can keep being informed about what's the latest trends or requirement.” Tier 1  “Because I accept infants, I wasn't sure for infants what I need to give them to eat and what amount is good for the children, for the infants, and I learned a lot from them.” Tier 2    “They give you guidelines of what ages need to eat what, and what is healthy, what's not healthy… not everyone is aware of things, let's say what chicken nuggets contain or what hotdogs contain, and what to feed and what not to feed the kids.” Tier 1    “The greatest benefit I want to say for me, again learning how to feed the kids healthy meals. Also, the trainings that they have those are very informative as well. I mean, we have to do a training once a year. Those are very good. I like those because there's always new stuff they come out with and also when they visit they come out and give you a new things you know, things change, where you can't serve this anymore or for instance, like the chicken nuggets, you got to have the CMA label now and things like that, which is which is good, you know.” Tier 1    “Another advantage is feeling like they have someone to call when they have questions around children's eating issues. So really, there's a whole nutrition education piece that ends up happening maybe more or less to begin with, but over time, things arise, and a parent wants this or that and they're like why and I can't do that.” Sponsor    “The benefit would be number one, the daycare provider learns how to provide and prepare nutritious meals for the children in her care.” Sponsor    “They learn a lot through this program of what may be considered healthier than other items. They also get to teach the parents good financial health for both the provider and the families and also provides a lot of learning what might be healthier for children.” Sponsor  “A lot of them learn a little bit more about nutrition. We try to go to the sites that we're reviewing and telling them about lower sugar content, cereals or yogurts, or the importance of collecting child nutrition labels for chicken nuggets or any other processed foods.” Sponsor |
| **Challenges of CACFP Participation** |  | |  |  |
| 4. Regulations | a. Burdensome daily reporting | | CACFP paperwork and daily reporting is burdensome and can be technologically challenging | “My hardest thing is they want me to put my foods... I'm very busy... sitting down and putting everybody into the food program, that's what makes it difficult.” Tier 1    “The difficulty of filling out the forms isn't worth it, in the end for the amount of money that you get back to our food.” Tier 2    “They [other child care providers] just don't think it's worth it for trying to go through all the paperwork and make sure that you're reporting every day.” Tier 2    “But if it's going to take me a lot longer to do, and it's more work then I question whether—I mean I'm already working 12 hours a day and it's just not worth it to me. If it's if it's hard to maintain, if I miss a day then I lose out on that day. It's a lot.” Tier 1    “Remembering to get it in each day, to get it recorded each day.” Tier 2    “It isn't difficult to do the menus every day [daily reporting]. But if you miss or you don't make it, that day is just lost [no reimbursement].” Tier 2    “We used to turn in paper menus and paper attendance sheets, and now that's not allowed anymore. So, there were a lot of people that don't have that kind of technological aptitude. They were like, 'Whoa, I don't want to do it this way.' So, they were really upset about that.” Tier 2    “In the beginning, it wasn't done on the computer. It was actually handwritten. When they started the computer portion of it, that was fantastic. But honestly, to have to do it every single day, it was more of a challenge than having to write down what I'm feeding the kids. If I could do it on a Friday before midnight for the whole week. Before would have been a lot better for me. But my challenge was doing it daily.” Tier 2    “You have to keep records and everything. That's the only hard thing because sometimes during the day I write what I serve, then at night I have to transfer it all to the computer. So, it's not the best, it's the paperwork.” Tier 1    “[The food program,] it's a lot of work. So, at some point, you have to evaluate if it's worth the time invested. So, I feel like we're constantly on top of things, we have to give the forms to the new kids, and then some kids expire, we have to send the expiration. And then every day, we have to do it that day or we miss it. And it's happened to me many times where I miss a day. And then that's it. It's done. I don't get it [the reimbursement].” Tier 2    “I know that lots of providers I know, they come out of the food program. They said too much headaches, too much paperwork, lots of things they have to do. The money, they are both upset, and they come out.” Tier 1    “It would be easier for reporting if I had more time than just one day to report, such as having the reporting open weekly, so that I could catch up on it, or even monthly to do it all at once.” Tier 2  “It's a barrier, you know, the fact that you have to do it on a daily basis, because if you don't do it daily, then you miss out on the reimbursement. So that's a challenge.” Sponsor    “Us asking them to do all that paperwork sometimes they just feel like that's not worth it for the money.” Sponsor    “Just needing to stay on top of recording meals every day, and enrolling kids, getting signatures, sending us that monthly claim. Just all little parts that are the nuts and bolts of being on it [the food program] so they get that reimbursement.” Sponsor    “Some of the other issues that we've been running into for providers as a barrier is just overall tech savviness… they have to engage their grandkids or their kids to help them and input their meals and snacks every day, and also sometimes we've found that that leads to discrepancies and misalignment between meals and children that were actually served versus what was recorded.” Sponsor    “A lot of the barriers that I hear from our providers come from the amount of work they need to do to get the reimbursement - keeping up with their enrollment, claiming daily, making sure they're in and out times are correct. We have them claim on an online system and some of them don't even use a computer or a tablet, so they're doing it off their smartphone. And so, looking at a very smaller screen compared to a large screen where we can see all the boxes, sometimes they miss things. So, I think for our providers, they would say the barrier for the program is first, using the online claiming system and having to do it daily and keeping up with that.” Sponsor    “I think they [providers] feel like there's a lot of rules, and we try to get them to stick to it and we do offer that online version for them to help them digitally. Maybe if the rules weren't so stringent on daily claiming, or they could go back [to claim at a later time].” Sponsor |
|  | b. Tier 2 requires more paperwork and sponsor support | | It is administratively burdensome for sponsors to support tier 2 providers with tiering determination; provider also struggle with the meal benefit forms. | “We do constantly try to look once a year. We always look at the data to see if we can make any changes [to their tiering]. And the other thing is we do encourage all of them [tier 2 providers] to distribute applications to their parents… It's hard though too, even doing that. Because some providers just don't feel comfortable giving out those applications… [they] don't feel comfortable, or they wait a little while until the parents been there a while and then they might offer them the application.” Sponsor    “This is a time consuming program. We're just getting to a place where… we can create our processes for supporting our tier two providers with the MBF [meal benefit form] process. Because obviously, we want to do everything that we can to support our tier two providers. If they can qualify for tier one reimbursement or for tier two high in any way, shape, or form, that is administratively burdensome. Especially since we have to verify every year, and if we do a parent MBF form and they're on a tier two mix, that's a whole other layer. It's just a lot of extra steps for tier two providers if we move them forward with the MBF process.” Sponsor    “Tier two providers right now are not interested in, like I mentioned before, the time it takes to complete the enrollment, or all the paperwork that's required per child from the provider versus her cooking whatever she has on hand and not dealing with all the logistics of the program that are required.” Sponsor    “Tier 2 providers, instead of doing all the paperwork that's required and it's not a lot of paperwork, but it is paperwork and time consuming. They'd rather just give the child whatever they can and not deal with the paperwork, and the visits, and everything that entails to participate in the food program.” Sponsor    “They don't feel comfortable asking their clients about their income and it's hard to explain to them that we have a letter that goes with the letter to parents in it explained to them that this benefits your childcare provider if you fill out this application [meal benefit form]… it's just a touchy subject.” Sponsor    “ Some providers get tier one and tier two because they have parent eligibility, or they have their parents eligible for their children to get tier one rates. So, you [providers] do have to separate all these things when you do your claim. So that is kind of a hassle especially if you don't have a program [software]… if you're a smaller program and you don't [have software] doing your claim is a little more work.” Sponsor |
|  | c. Nutrition standards | | Meeting the CACFP nutrition standards and meal pattern requirements is difficult or there is difficulty finding CACFP eligible foods. | “The greatest challenges are making sure we have a huge variety of different types of foods and different types of things for the children to try.” Tier 2    “Sometimes [it’s hard] to find 1% milk, for example. To see that it is not in all stores, like Costco, there is no 1%. Then how do I buy in quantity? I have to go to another store.” Tier 2    “Just keeping up with the changes in the quantities or types or the milk for different ages has changed. Just keeping up with that.” Tier 2 provider    “I've had parents come to me and say, “Well, my pediatrician said that I should continue to give whole milk,” and I go “I know, but it's a food program requirement. So, there's nothing I can do.” Tier 2    “The typical way [that provider serves meals], it's all family style. We put it on the table, children serving themselves... And then we learned that, from the program… that will not guarantee each child will have a given portion of it. Because if they serve there's no way the teacher would measure, to essentially have these children taken certain amount of food.” Tier 1    “It's really a problem [strict regulations] , the milk is a huge issue right now. We need options, if we had options, this whole issue would go away.” Sponsor    “One of our biggest struggles is the milk. It's the issue that they have with serving so much milk. The other one is the fact that they have to serve a specific type of milk. We have a lot of issues when it comes to reviews with the milk, they either have the incorrect one, or they don't have enough of the correct one, whatever the case is.” Sponsor    “With our meal pattern, especially with lunches and stuff, you have to serve one vegetable and a fruit or you can do two vegetables. They struggle sometimes because they don't like to waste food and because you have to put everything on the plate, they feel like they're wasting so much. Same thing with the milk. And then the other thing is, they feel like the kids gravitate towards the milk and they gravitate towards the fruit. So that's all they want to eat. So, they want to be able to give them their meal, component by component kind of, so that they will eat it all. A lot of our providers struggle with that. Because they're like, "you know, I've been doing daycare for X amount of years and this is the best way for me to get them to eat their meal." And yet I'm coming [the sponsor] to do the visit. I'm saying you got to serve it all together and you can't withhold components until the end. So that's been a struggle.” Sponsor    “I think one of our biggest struggles is the milk. It's the issue that they have with serving so much milk. The other one is the fact that they have to serve a specific type of milk. We have a lot of issues when it comes to reviews with the milk, they either have the incorrect one, or they don't have enough of the correct one.” Sponsor    “Program requirements are just too rigid, too much. They don't understand that we can't give handouts, that we can't just say like, "Oh, it's okay, that you didn't have the milk will still pay it". They get frustrated after years and years of having to follow same requirements and the reimbursement not being enough and now with higher costs of food, they just figured it's time to not be with the program.” Sponsor    “It seems like there's a lot of rules and especially the food rules get real picky.” Sponsor    “You know, the program requirements sometimes are a little rigid. So, any disallowances, they add up really quickly. So, it can be really difficult when you see that they're trying really hard to meet every need or every program requirement, but they [providers] fall short with one component, and yet the entire meal gets disallowed. So that's probably one of the hardest things.” Sponsor    “If they [providers] have the wrong milk then they're you know, deducted the meal and they have to deal with the little stuff like running to the store when it's very inconvenient because they ran out of something. Or they bought the wrong bread, it's organic, it's not wholegrain.” Sponsor    “It varies from provider to provider but a lot of it is that the regulations seem to be very strict for them. Specifically following the mealtimes. A lot of them share that things happen with the daycare that are kind of unplanned and they get behind… managing time and also ensuring that they're following the regulations around meal time.” Sponsor    “I had a provider who was doing a great job and serving all her five components in her daycare for lunch and then when I was checking them out to see that it was the correct milk, she was confident enough that she had the correct milk, and it ended up being 2% milk. There was no way of replacing the milk in that moment, and so in that moment, we did have to disallow because she didn't have that milk component correctly, and there was no option to really provide immediate response to make sure she does get reimbursed. And so, we disallowed her, and she just said it is kind of unfair because all the items are there except for the milk. So, for that one single items, she's going to be disallowed for everything. Four out of five items were there and then it's such a great loss because they have other items that cannot take the cost to put on the table and it can be a little frustrating for them.” Sponsor |
|  | d. Monitoring visits | | In-person monitoring visits are challenging. | “I enjoyed having Zoom calls because when somebody comes in...it kind of causes a little bit of chaos in the group because somebody new is here.” Tier 1    “Some providers don't want visits; they don't want us to come unannounced out to their house. Which I get but they're from their perspective, It's you know, "I run my own business. I'm a businesswoman. I started my own business so I can do things my way. So, I don't need you to come tell me what to do." Right, these are strong women, they started their own business, and they have ideas of how they want everything done. So, when we come in unannounced to do visits, it disrupts their day. It also, we're saying you have to do this when they really want to do it this way, and we're saying but in order to get the reimbursement you have to do it this way. So sometimes it just doesn't mesh well for them.” Sponsor |
|  | e. Serious deficiencies | | Serious deficiency hinders CACFP participation, is perceived as being “too harsh”, time-consuming for sponsors, or creates language inequities. | “If you're [provider] going on a field trip, technically, you're supposed to notify the office that you're going to be away... you're busy trying to get everything organized and that might slip your mind… you're sent a warning letter saying that you didn't follow the regulation… if that were to happen again, that puts you into serious deficiency. And if you're terminated from the full program, then you're not eligible to claim from the CACFP if you're placed on the National Disqualified List for seven years. I think that those regulations are a little too harsh… seven years for not calling the office. I think that is a little too severe.” Sponsor    “We also see that with the language barrier too… if they don't speak and read English, and are also not tech savvy, they'll usually have a partner or someone else do it, which again, leads to those errors and non-compliance.” Sponsor    “The way that process [serious deficiency] is set up is very not user friendly. It's very subjective. It's not clear. It's difficult. It's time consuming, and it really needs a rework… as a sponsor who has to take those steps and administer those processes, it's not good. It's not a good process at all.” Sponsor |
| 5. Inadequate reimbursement | a. Tiered rates are inadequate and unfair | | Tier 1 and 2 reimbursement rates are inadequate to cover food costs and not worth the effort. The tiered system is disliked and perceived of as unfair; providers recommend a universal tier. | “[The tiered reimbursement is] not fair. We all should be on tier one.” Tier 2    “I personally think that we should all be the same [tier]. We're all doing the same thing. We're all serving the same thing. So just because you live in a different area, I don't think you should get less.” Tier 1    “I really wish that everybody could be on the tier one. I feel like everyone is paying the same amount for the food regardless of whether they are low income or private pay. To provide healthy meals for the children, everybody needs to have the same equal opportunity. You wouldn't want a child to be fed less because they're one place over another. All the children should have the same opportunity.” Tier 2    “All children, regardless of their social status, they deserve to have healthy meals. By providing the tier one, everybody has the same level playing field opportunity. Just because a child is not tier one doesn't mean that the family doesn't cut back on providing healthy nutritious food for them. And by putting it on the provider at the lower rate during an insanely inflationary time, you're really making that provider have to choose between providing the healthiest food for the children on less money that doesn't go as far and it's really not fair. Every child should have the opportunity to have a wide variety of healthy, whole-grain, whole foods. And with the higher reimbursement rate it makes that more possible.” Tier 2    “The tiers don't make sense. They should be all the same, and they should be all at the highest level that they can be. The point of the food program, my understanding was especially for kids that are subsidized. And since most of providers are low income themselves, and half of our kids are low income, why not provide them with as much food as they can get, just so they can stay healthy.” Tier 2    “I don't think that there should be two tiers. I think one tier, it's already not enough to cover the cost of food. So having two tiers just makes it doubly hard for those folks that want to participate but it isn't worth their while to do it.” Tier 2    “I don't really agree with that. I don't think that there should be a tier all kids are equal. It doesn't matter if you come from a low income family or middle class or upper class. Everyone is the same, we eat the same food. So why are we tiering kids at different prices? I never understood that.” Tier 2  “I just don't really know why they tier different kids. That always has bugged me. And I've been in the food program for a long time, since I started. I just don't know why you can tier people. They're all the same. It shouldn't matter what their parents make. Everybody eats the same food.” Tier 2    “The two tiers, if I were a lower tier care provider, I might be a little not happy because of where I live, even though I have to serve the same amount, the same foods. So, I think maybe looking at that, why there has to be two tiers where one provider gets less than another when they're doing the same exact thing.” Tier 1    “There is tier one and tier two. I always feel this is really not fair. Because if I live in that zip code, I buy the food the same like a person, a provider, who live in a different zip code. It is the same prices. … So I'm required to serve the children a healthy meal, whole wheat, fruit, vegetables, with pennies... The payment is really a challenge. We went to Sacramento to advocate for that. I advocate with our R&R. I advocate everywhere, where I said, "We need to be, all of us, equal on tier one, because it's already pennies." Even tier one will not cover the meals; I have 12 kids.” Tier 2    “For me, because of the area that I live in, doesn't necessarily mean that I should get paid less for feeding kids versus somebody that's in a low-income area. I understand it, but I don't necessarily agree with it.” Tier 2  “I feel like it should be equal… it’s not fair [tiering] because the food and stuff that we purchase and the kids that we take care of have nothing to do with it.” Tier 1    “I do know. It's been very nice the last couple years with COVID not having the tiered reimbursement. I did get notification that was coming back. So, I'm not sure what tier I'm on, that's part of my confusion with that. I know in the past, I had to qualify for tier one. I think it was tier one. That part's a little confusing. I wish it was just, "Across the board, here it is, it's what you get." That would be so much easier than what it is.” Tier 2    “To me I wish we would all take one tier, and if it could be the highest tier is good because some people are not wanting to you know, take part in the food program because of the lower tier. The lower tier, they don't make more so they just don't want to do it. So, for me, I feel we should all be under the same and we should maintain the highest tier the tier one for everybody so that it will encourage people to do it. Now that food prices are very expensive.” Tier 1    “I think it should be equal across the board. Because even if we're not in a low-income area, the food actually might cost more than it would going to different neighborhoods. The cost is still something that we're taking on to try and provide the healthy food. So, I feel like the reimbursement should be more in line with what we're spending instead of where we live.” Tier 2    “I don't like it at all [tiered reimbursement rates]. I feel it should be the same for everybody. I don't qualify for a tier that pays more money and I'm just barely above it. And three quarters of my children are low income, but because they don't fill the paperwork out, I'm not eligible to be reimbursed for the kids that won't fill out the paperwork. I think everybody should have the opportunity for a quality meal.” Tier 2    “I think it should be one tier. I've always thought it should be. I don't understand the logic of having multiple tiers because, if it's the reason I heard, and that's, "Oh, well, you're in a higher income and so you can charge more..." That's just not true. I have low income people that will come here and drop their kid off because it's a nicer area or it's a nicer school district. But that doesn't mean I can charge them more. I keep everybody on one price level. I think it would be easier for the USDA food program to deal with it, it's easier for us to deal with it, it's easier for budgeting, and it puts more money in our pocket to spend on the children and the families.” Tier 1    “I just think everybody should be on one tier. I don't get the two tiers. I think it needs to be one tier across the board. I don't understand the justification of making it multiple.” Tier 1    “It will be nice for everybody to have the same tier reimbursement, especially since tier 2, even though they live in a more affluent area, that means they pay more for food and they get reimbursed much, much less than what tier one gets reimbursed.” Sponsor  “I think that the tiered reimbursements should be back the way it was 24 years ago, 25 years ago, or more than 25 years ago. Because the cost of food is still the same for every provider, whether they live in a high-income area or low-income area. And a lot of the providers that have children in their care, they're charging what they can whether the child is low income or high income. So, if we were to get that tier one rates for everybody across the board, it would encourage providers to keep on the program more, more so than not.” Sponsor    “I think it's the most unfair thing I've ever heard of. What I see is providers don't make a lot of money no matter where they live, and if they live in a higher income neighborhood, they're probably spending more on housing and more on food. So, they're just making less…If tier one already is getting very little money, let alone tier two, they get cents to the $2 that the tier one gets reimbursed. And that is very upsetting, very demeaning to be told, “Because you're in a more affluent area, you get less.” Sponsor    “We all shop at the same stores. Regardless of where you live, especially low-income areas, a lot of them don't have grocery stores. So, a lot of times they're even paying a little more for their food because they don't have the big grocery store chains in all their neighborhoods… So, it's not really fair that because of where they live, they're getting less. They're all serving the same clientele, just because you live in a higher income area, are you getting higher income kids, it doesn't work like that. A lot of times it depends on where parents live themselves, where they're going to school, where they're going to work, where their spouse works… And it's not always based on area, so it's a little unfair that it's based on an area. I think providers would love it, if everybody got the same rate. It's just unfair, and it's not always based on where you live or your area that you're going to be servicing clients that are higher income, it doesn't always work like that.” Sponsor    “I don't think anybody should be reimbursed any different than anybody else. Just because you're in a certain area, you're still buying food, the cost is still the same. Why should your reimbursement be lower than somebody that lives somewhere else?... I've always felt that everybody should get the same amount of reimbursement. I mean, food cost the same for everybody, doesn't matter where you live, what area you live in. So, reimbursements should be the same.” Sponsor    “It is a real benefit to really consider either just eliminating the tiering and raising that reimbursement rate to a standard that will really help these day care home providers in their mission to give nutritional, quality meals to these kids.” Sponsor    “I believe all providers should be treated equally. Food costs the same everywhere. It doesn't cost any more at one particular grocery store because of the area you live in. And I feel that it's important to treat all children equally and it's important for them to all be able to have access to the same nutritious meals whether you're tier one provider or two provider…. tier one and two tier, it's just a hindrance. I think that all providers should be paid equally, no matter what.” Sponsor    “I feel that providers who are in the tier two regions should also benefit from tier one and to align with what school is doing [universal meals for all K-12 students]. Because one of the disclaimers in CACFP is that this program should be offered to all children, regardless of race and ethnicity. The program has been offered to everybody that the providers are able to reach. I think that because it's offered to everyone equally that providers should also receive the same reimbursement. Those that are in tier two should get tier one. We don't have a lot of tier two providers and mixed tier providers in the program. A lot of them get very discouraged to find out the gap between tier one and tier two. It's a big difference.” Sponsor    “I think that we should all be the same [tier]. We're all doing the same thing. We're all serving the same thing. So just because you live in a different area, I don't think you should get less.” Tier 1  “[Inflation] has impacted my ability to provide food for my family, because I'm having to dip into my own family's budget for our groceries to pay for food for… for the children that are in my care that I'm lawfully required to provide for.” Tier 2  “The challenge has started the last couple of years when the food became really expensive, like tripled prices, everything is going so expensive.” Tier 2  “The prices have increased a lot for food and with what they reimburse us, we don't buy the same anymore. We buy less food with what we get reimbursed, but we have to put more money out of our pocket.” Tier 1    “Yeah, all expenses have gone up. So, it's gotten to the point I can only raise my prices so much and the overhead is definitely impacting. I'm making less income than if I would a corporate job. For many years now I've borrowed so much money just to stay open.” Tier 2    “Yeah, the prices have been getting a little higher, so for some of the meals, a lot of us had start going shopping at the Dollar Tree, the salads and the veggies. Because everything is so expensive. And we've been buying lots of cheaper stuff.” Tier 1    “Our money doesn't go as far and we're having to cut back wherever we can.” Tier 2    “The value's inflation, food cost and gas, everything is directly connected to my cost. From last year to this year, the cost increased a lot that means the profits are coming down. So even my family and I started thinking, "Why do I even need to do the business when I'm not getting enough money?" So that is affecting the entire business and not only the food.” Tier 2    “It definitely impacts, because inflation, everything is more expensive… To make the food I used to buy, $20, now it's $45, the same thing. Every time you go, it's $2 up, $3 up or $5 up… It's so expensive. And of course, it impacts us because we have to pay it. I pay it from my own pocket, then I get less on my income. I buy the food that I'm supposed to buy, I bought it no matter what. But then I became less on my own bills, I'm short on my own bills.” Tier 1    “It's made things tighter as far as spending money. "Do we really need that?", as far as that food this week? Or "No, no, we need to save because we have to buy groceries or whatever we need." Everything's just a little tighter these days.” Tier 2    “Pre all of this, I would spend $400 a week on groceries and now it's $600 a week. So, it's some additional $800 a month more that I spend in groceries than I used to. Financially, I'm not making this much. I'm looking at needing to raise my rates because with everything going up and having to pay employees and food going up. You know, I'm not doing as well as I did when it wasn't as expensive.” Tier 1    “I really have to budget now. I have to take kids to school; I have to buy more food. More kids, more food, more expenses. It's impacted a lot.” Tier 1    “It has impacted my ability to provide food for my family, because I'm having to dip into my own family's budget for our groceries to pay for food for them. So, my family's meal plans are suffering because I'm having to make sure I need the bare minimum for the food program, for the children that are in my care that I'm lawfully required to provide for.” Tier 2    “Just there again, prices of food these days, everything's more expensive. You go to the grocery store, spend $100, and you have one bag of nothing. When you're feeding six to eight kids a day, it gets expensive.” Tier 2 |
|  | b. Inadequate sponsor administrative funds | | Sponsors perceive the CACFP administrative funds are inadequate. | “I think the challenges that we have is that we need to get more administrative funds per home that we sponsor so that we can hire more staff to help us do our end of the work too. That's the biggest challenge that I can see.” Sponsor  “The admin rate isn't sufficient to cover the cost to run the program... I don't think that this program is designed to have full time workers with benefits. It's just not set up with enough funds coming to admins [administrators] for that.” Sponsor |
| 6. Food waste |  | | Children do not eat or drink all the food and beverages, resulting in food waste. | “Yeah, the milk is hard to provide, it's wasted so much. So, it feels so wasteful and as expensive as it is I try to buy as little as I can just enough to meet the criteria. My daycare kids don't really like the milk. I've also tried almond milk and other milks to see if they prefer that, but they just don't. But it's definitely expensive and so I waste a lot of it and it's a big frustration for me... I just had to throw out half a container the other day because it went bad because they don't drink a lot of it. So, it's hard, that one's hard.” Tier 2    “I throw away a lot of food... it's hard to get little kids to eat broccoli... I offer it to them every day, but more likely than not, they don't eat it... a lot of times the vegetables are hard to get them to eat.” Tier 1    “They [other childcare providers] feel like they waste so much food because they have to put it in front of the child even knowing that the child won't eat it. They have to put it in front of them. They feel like it's been kind of a wash and so they've quit the program.” Tier 1    “By strictly following that guidance [CACFP nutrition standards], there was a lot of food waste.... you put all the meal before the child while the child may not finish it. What are you gonna do with that? Throw it away while you also understand, how many children are actually really hungry for food? So, I think that's a huge waste of food.” Tier 1    “We hear a lot of them say "The kids don't like the vegetables so why would I put it out because they're not going to eat it.” Sponsor    “They struggle sometimes because they don't like to waste food and because you have to put everything on the plate, they feel like they're wasting so much. Same thing with the milk.” Sponsor |
| **Family Perceptions of CACFP and Child Care Meals** |  | |  |  |
| 7. Awareness of and appreciation for CACFP |  | | Families are aware their provider participates in CACFP and may express gratitude for the program. | “I didn't really know it was a thing until she had told me that she was part of the [food] program and that it was provided.” Family  “I don't know too much about it [CACFP]. I just know that they're provided the food and that includes the two snacks and the breakfast and lunch.” Family  “That I believe she gets like $5 a day per kid [from CACFP] to provide food, snacks, all that kind of stuff... we are a big fan of it. It was not something we were familiar with before starting with our provider... it's a really nice perk. We like it. We like participating in it.” Family  “I didn't even really realize how amazing and lucky I was that she was part of it [the food program] because it is such a struggle for me sometimes to make sure I have lunches packed in the morning. It's not something I've ever really had to do in the past... being a busy working full time mom, I don't have to worry about packing the food and also, it saves us financially. I know it's saving us money as well. I don't really know the details of the program, like the why or how, but I just know that I appreciate it... I feel like blessed in the sense that I know, it's been something I haven't had to worry about in the last few years. I would hope that other moms and children are getting that because I know that even though we live in this first world country, there's the food shortage and kiddos going to school hungry and not having snacks and stuff. I just find comfort in knowing this is not a concern for my little and all of her friends at school [daycare]... I didn't realize how lucky I was that we had it until my little didn't have it anymore. I feel very grateful for it.” Family |
| 8. Importance of meals/snacks offered with childcare | a. Families value childcare meals/snacks | | Families indicate healthy, high quality, and a variety of meals and snacks are provided to their children and that this is important to them and may be a consideration for selecting a childcare provider. Providers and sponsors also indicate CACFP has a positive parental perception. | “With the economy changing and everything going up in price, it is hard to pay child care and then pay food as well. It's just an additional costs that comes out of your pocket. And it's nice when it's included [in the cost of child care] because that is one less thing that I have to worry about...” Family  “I've had a daycare before where they didn't tell me that they did that, so the whole time my child wasn't eating, and I didn't know that until a few weeks later. But that's when I decided to switch to the one [daycare] I'm at now...” - Family (AFKBFA)  “Yes [it is important]. To make sure they get fed.” Family  “Well, at the age that she's in, when she'll eat almost anything, I'd like her to get as much benefit from the food that she's getting [in childcare] as possible and hopefully set her on a path towards maintaining that type of lifestyle... As much as we can have or eat things that are more natural, not processed. But at the same time, we believe in moderation. So, it's not like our kids don't get treats or candy or any of that kind of stuff. It's just our preference would be that they have the healthier, more natural version.” Family  “I feel like it's really important [healthy meals and snacks in childcare] because by the time I get home, I'm really tired and I'm ready for bed. And it's good to know that they already had that because I didn't have time to do it. She's [childcare provider] kind of helped me out with that. I think it's really good... I feel like it's [quality of snacks] very important because I would want them to have fresh food. I feel like nobody wants to eat food that's not fresh... It's [variety] very important because I don't want him to be picky. I like him to explore his options with food and not just stick to the same meal. ” Family  “It’s important [healthy meals/snacks]... very important... I would trust that for the most part everything that I'm aware they're eating is nutritious in some way. And so, that to me is more what's important... Sometimes my child loves apple and then all of a sudden, she won't eat apple anymore. She's been served different fruits, so, I just want the exposure of continually being served the nutritious food, even if it's not something she's choosing to eat. I have had conversations with my provider about that where I'll say like, "Oh my gosh, is she eating apple for you anymore?" My provider will be like, "Oh, she maybe had a few bites of it with breakfast, but at lunch, I served this and, and she ate all of that." So yeah, I feel the ability to still get the nutrition and still get the benefits of the healthy food, so the exposure to different foods is important. And I know that that's happening there.” Family  “[It’s] very important [healthy meals/snacks]. I think healthy is the first thing. Healthy to be healthy.” Family  “I think it's definitely important that children try any and all foods, especially vegetables... fresh vegetables are very important in being able to try those, you know, new vegetables its good because it broadens the nutritious and they may be getting things may not get at home, too, so it's good to see that that is something they're getting. So, they may be trying things that I don't serve at home. I do try to, you know, try different fruits and vegetables and just try them with my children. But I think it's good for them to have a variety of different foods.” Family  “The kids are eating healthy foods. It's a good way to get parents into my daycare because I do feed them healthy foods.” Tier 1    “Once my parents know that I am with the food program it does make them feel more comfortable because they've had a daycare before, and all the kids got was peanut butter sandwiches and water... some of the people don't know about it... I do tell them that I am on the California food program and that people come through and they inspect and that we have to make sure we have a protein and bread, and that we have to serve grain and vegetables and fruit and they're like, "Oh, that's really nice!" I think it makes them feel better because they have had a lot of bad experience.” Tier 1    “Parents, I feel like they look at it in a good way, as far as knowing that you're in the food program, that the kids are not just going to be eating lots off junk.” Tier 1    “I convey this message [CACFP participation] to my clients, and they have a lot of confidence that the kids are eating healthy food, and somebody is supervising and guiding.” Tier 2 provider    “I think it's more the variety that the kids choose to eat and being able to tell the parents… because I send out menus every day of what they've had. And they'll say, "Oh, they don't eat that for me. I can't believe you got them to eat that!" Or, "Oh, they've never tried it!" So, the parents get more involved in it.” Tier 1 provider    “The marketing piece of when interviewing parents that you have the opportunity to tell them that they don't need to bring meals, they don't have to pack lunches, they're already going to be getting healthy meals with oversight from the food program, and so that's, to me, that's a big marketing advantage that some of them don't realize.” Sponsor    “I always hear that kids come into the daycare and their parents are like "Oh, they don't eat the specific food" and then after they've been with the provider, they're very shocked how open and willing they are to try new things and now they integrate that at home.” Sponsor  “Parents, I feel like they look at it in a good way, as far as knowing that you're in the food program, that the kids are not just going to be eating lots of junk.” Tier 1 |
|  | b. Negative impacts anticipated if provider stops offering food | | If providers stopped offering meals and snacks as part of their care, this would result in challenges to the families and may result in them changing childcare providers. | “It would probably make it to where I would need to look at another childcare provider. Because that would be an extra hardship to have to do that on top of all the other things and childcare costs, and all that sort of stuff.” Family  “It impacts me because I still have to buy groceries for my house and it's going to be hard to do it every single day over there as well. So, I still have another kid to feed. It's just going to be harder.” Family  “Cost will be increasing for us.” Family  “It would probably make it to where I would need to look at another childcare provider.” Family |
| **II. Perceptions of CACFP During COVID-19 Waiver Period (Higher Reimbursement)**  These themes and subthemes reflect perceptions of CACFP when the tiers were waived, and the impact of the temporary higher reimbursement and subsequent reinstatement of tiers from interviews with providers and sponsors. | | | | |
| **Theme** | | **Subtheme** | **Description** | **Example Quote(s)** |
| **COVID-19 and Inflation Challenges** |  | |  |  |
| 9. Difficulty finding CACFP-eligible food |  | | Pandemic supply chain issues and inflation impacted providers’ ability to find CACFP-eligible foods. | “The biggest challenges... right now [is] that food [cost] has gone up a lot… to be able to provide good food to the children and that we can afford...” Tier 1  The greatest challenge has been when COVID hit me, and we needed to provide milk. And there was no milk in store. So that greatest challenge was, you know, 2020 2021 when we had the COVID and there was no milk in store. But we had to provide milk, so that was a challenge.” - Tier 1    “One of the main barriers is basically access to food. During COVID, a lot of our daycare providers had a hard time getting a hold of, I think mainly milk was an issue a lot of people shed light on how that was not attainable.” Sponsor  “The biggest challenges... Well, right now, with the inflations, that food [cost] has gone up a lot, this is the main challenge - to be able to provide good food to the children and that we can afford the expenses.” Tier 1    “To be honest, after the inflation, the prices [reimbursement] should go higher. I'm not going to say cover it 100%, I wish, but it should be reasonable with the prices at the store. You cannot give us $0.10 and expect us to serve two meals on one snack.” Tier 2    “It's very hard with inflation and the food [prices] going higher, and the USDA saying we [have] to do more whole grains and stuff like that. Anything healthy is gonna be double the price, that's just how it is. So, I try my best to do the fresh fruits and the whole grains. And we do, we make it, but it costs much more than it did.” Tier 1  “I used to go to one store to purchase everything from there. Now I go from store-to-store with the newsletter to check what's cheaper. That Safeway or compared to Lucky? Where should I go to buy this or this? So, it's more time preparation at home with my list.” Tier 2 |
| 10. Negative impacts on food and business budget |  | | Providers indicated negative impacts to their business and food budgets due to COVID-19 and inflation. Sponsors observed providers choosing cheaper, frozen, or packaged food items due to budget constraints. | “We are in a crisis. I have to keep them [other staff] employed, their salaries. I have to keep the parents [the] same tuition. And I'm the one who takes the cut from my paycheck, always.” Tier 2  “I put together fresh fruits and vegetables, but now because of the cost, I'm trying to get canned foods and vegetables or the low-cost fruits and vegetables, especially the high end, good quality berries and cheese. Those kinds of things are definitely gone from my menu now.” Tier 2  “It's really impacted labor. People don't want to work because the cost of living has gone up. And my employee expenses have gone up over 25%, just to keep an employee.” Tier 2    “We're having to buy more items that are less of the convenient [items] that are ready made. We're having to make stuff from scratch more and put it together, so it's taking quite a bit more time. And that's part of the reason my hours had to be shortened because I need time to be able to do all these things.” Tier 2    “It was a crisis in our field, paying the assistants. I cannot raise the prices on the parents…. the provider is suffering. What I pay for my assistance, I cannot make it less because they will leave me and go to work at McDonald's— they pay more—or at Starbucks. We are in a crisis. I have to keep them employed, their salaries. I have to keep the parents [the] same tuition. And I'm the one who take the cut from my paycheck, always.” Tier 2 |
| 11. Decreased child enrollment |  | | Child enrollment at family childcare homes decreased due to COVID-19, leading to decreased CACFP meal/snack claiming. | “My income was going down from losing lots of kids and the pandemic made income very unstable with getting kids and keeping them and then also outbreaks and such.” Tier 2    “When the pandemic hit, I lost every single daycare kid. All my daycare kids stayed home. I went from having a full-time job. It was 10 hours a day, five days a week, getting paid to not getting paid. I lost all my kids.” Tier 2    “During the pandemic we lost lots of our kids. And we lost the food program money, because we don't have the kids.” Tier 1    “My enrollment went down when COVID hit and has never come back up to what it was pre-COVID.” Tier 2    “With the pandemic, some people closed their daycare as there was a big panic going on so, some of them closed. We did lose a few in the beginning because of that.” Sponsor    “I think because of the pandemic we were losing providers because they didn't have children, or they didn't want to stay offering childcare because they were older, and they didn't want to expose themselves to COVID. So, we lost a lot of providers on that end and that affected our claiming, versus how much they were getting paid.” Sponsor    “A lot of daycare providers have dropped from our program due to, well, it affected a lot of people. With COVID, parents lost jobs so they you know, they couldn't afford the daycare.” Sponsor |
| **Facilitators to CACFP Participation During COVID-19** |  | |  |  |
| 12. Removal of tiered reimbursements | a. Supported enrollment and retention | | Encouraged new and existing providers to join or stay on CACFP. | “I first got on the Child and Adult Care Food Program because they had told everyone that they were going to make everybody tier one due to the pandemic and so it seemed like a good time to get back into it because of the fact that my income was going down from losing lots of kids and the pandemic made income very unstable with getting kids and keeping them and then also outbreaks and such.” Tier 2    “It takes a lot of time to do all this stuff. And the higher reimbursement rate made me want to stay in the program.” Tier 1    “Having a higher rate impacted me by helping me keep families on the food program. It helps reduce costs for the families I serve, since I serve low-income families. And it helped reduce my own business expenses.” Tier 2  “The higher reimbursement rate made me want to stay in the program. Whereas the lower rate… I just questioned whether I wanted to do it because I wasn't making all that much money on it anyway.” Tier 1    “We saw a huge increase in the numbers of providers that got back onto the food program and got back on purposely for that. A lot of us that weren't on the program. Part of the reason I wasn't on it was because there was a lot of paperwork. That was before it was online, because now it's online. Now it's a lot easier. Also, it's just better for everyone to be on that same tier. A lot of people really wanted to come back to that program.” Tier 2    “It was huge for me, when I remembered to claim it… Having that extra income, even if it was just double what my normal was, was great. Because we ate lunches, we ate snacks.” Tier 2    “In general, it was incredibly helpful when they provided the higher COVID rate… because I won't compromise my quality of care… it was definitely helpful so that I can stay open so that I can operate the other aspects of my program with the same budget.” Tier 2    “You look at our numbers, you know, when the pandemic hit, I mean, when tier one for all came, everybody joined.” Sponsor    “It helps rise the numbers of providers that were participating in the food program.” Sponsor    “We grew because all of the providers that were classified as to tier two providers heard about them being reimbursed equally. So, we grew, not by many providers, but we did grow in our tier two areas.” Sponsor    “The tier two providers are much happier because they were receiving the higher rates per meal per child versus what they were receiving before.” Sponsor    “I thought it had a clear impact on providers. I think for the most part it really encouraged providers to remain actively participating in the program… The reimbursements were an extra benefit that they [providers] saw, and it just reminded them that all their work is paying off. I do think that it did cause for providers to stay longer [on CACFP] and be active and be open during COVID… They would share that it's nice to see that amount is a little bit higher because it helps them remain open and during those during that hard time.” Sponsor    “We lost a lot of providers at the start of the pandemic and then they were coming back, and our numbers were going up and up… I do believe there were providers that just never started their business again because of when the pandemic started, but we still had that nice dangling carrot out there [higher reimbursement]. And so, we were able to continue growing and we got close to our pre pandemic numbers.” Sponsor    “We gained more people who were in the tier two areas [geographically] because they were getting that higher single reimbursement rate [tier 1]. That's how we could market it to those areas, where now because of the pandemic you could get the higher rate so you should participate and start to be in the food program.” Sponsor    “I think it encouraged it [higher reimbursement]. I think that was really an opportunity to reach out to daycare home providers that wouldn't normally participate because of the rates. Our tier two people were very excited to be receiving more of the reimbursement.” Sponsor    “It was very encouraging for them [providers]. It gave them some incentive to get that paperwork into us, get those kids enrolled, and let's get started because once you start getting the reimbursement it's a lot easier.” Sponsor |
|  | b. Offset inflation | | Higher reimbursements balanced out rising food costs due to inflation. | “It [higher reimbursement rate] made me able to still be able to provide home cooked meals for the kids because the cost of food has— where I live—the cost of food has probably doubled and maybe tripled on many items.” Tier 2    “I feel that the food costs have gone up faster than my rates. So, then I joined the food program a little after I opened and then that worked out and I was like, 'Okay, I'm within my budget still.' And then COVID hit and then the budget went up. And I was like great, because food costs are going up.” Tier 2    “Everything was high anyway and in demand, so it didn't change a big deal, even though I had eight kids at the time. It didn't make a big difference, because everything was expensive, and it still is.” Tier 1    “It helped out, the more investments you get, it helps. You can pay for the food; you can pay for everything you needed.” Tier 1  “The lower rate is just not even close to helping cover between the meal prep and the time put in… And when you get $250 it's like "Oh!" You're kind of busting your butt. But when you're getting $700 or something, it made it much more reasonable, it covered a lot more.” Tier 1    “Definitely, food prices have increased. I think the lower rate pre-COVID was still not quite enough. And so definitely, the COVID rate was a big help, but food just keeps going up. So, it helps fairly until food goes up, again it's skyrocketing.” Tier 2    “Hopefully this reimbursement at the higher rate will help you do that, but I think because inflation was going up really high it wasn't catching up. They still felt like it wasn't enough.” Sponsor |
|  | c. Better meals/snacks | | Some providers indicated that the higher reimbursement resulted in improved quality and variety and increased the quantity of foods/beverages served, and made it easier for providers to shop for food. | “I was able to provide more variety of fresh fruits and vegetables with the higher rate.” Tier 2    “The quality changed. With the lower rate I was doing more canned fruits and vegetables, and with the higher rate I'm doing more fresh fruits and vegetables.” Tier 2    “I feel like you're able to buy more fresh fruits and vegetables versus canned, because you can buy canned by the case and it's a little bit cheaper. Whereas fresh fruits and vegetables are more expensive.” Tier 2    “I did start buying some different-- I bought more proteins, more vegetables and more fruits at that time.” Tier 1    “Having the extra funds for fresh fruits and vegetables versus canned fruits and vegetables was huge.” Tier 2    “What changed. For example, I could serve blueberries… I could serve more chicken. So yes, it's changed with fruit with the vegetables, I could go and buy different kinds, several kind.” Tier 2    “It allowed me to be able to pay those prices for fresh because I'm a person that I like to use fresh foods. … So, it did allow me to be able to continue to buy the fresh produce.” Tier 2    “We were able to give them more brown rice, more whole grain pastas and a lot more higher quality foods. We were able to give a whole wheat bread at the higher level quality organic one, as opposed to a non-organic one. Different brands that tend to have a lot less preservatives and things in them, and they're more whole foods, less ingredients. Tier 2    “I would say the quality did increase with the higher rate. The quality and variety both increased with a higher rate. I was adding more whole food meat, like chicken and ground turkey, I was cooking with more of those foods, versus prepared meats like deli meats and chicken nuggets and hotdogs… I was able to offer more and seconds with the amount, with the higher increase. And with the lower rate, I was serving the amount that qualified for the food program but wasn't able to offer as many seconds.” Tier 2    “Higher reimbursement rate, yes. I used lots of eggs and high-quality cheeses. And thinks kids like, like string cheese and cheddar cheese, those kinds of foods.” Tier 2    “With the higher reimbursement rate, we were able to buy a larger variety of the different types of protein. We were able to give them chicken, turkey, fish, eggs. There was much more variety.” Tier 2    “With the higher pay, you definitely get better quality… if I get more pay, I give them more variety.” Tier 1    “I was able to buy a more, different variety of the whole grains just to get them something to try instead of regular honey nut cheerios, they actually have a whole grain honey nut cheerio. And it's a little more expensive but I definitely tried to buy that one… it's just a little bit better for them a little healthier for them.” Tier 2    “It was sufficient. My students that eat, I was getting more options for them giving them expensive fruits and vegetables than just banana and apple. So that directly impacted the children and their intake.” Tier 2    “It was very helpful… I could get more of the organic stuff; we would go to the fruit stands quite a bit and get organic fruits and organic vegetables. I was able to take it a notch higher, in my opinion, to make it even more healthier and I guess maybe give them more of a variety of things.” Tier 1    “When it was going higher, it was going to help us a lot because at that time my husband didn't have a stable job and then everything gets harder... And when I got this food program and every time when I got their reimbursement, I felt kind of a relief. So, I can have this kind of money to spend on fruits and vegetables and for meats.” Tier 1    “The higher rate reimbursement helps me keep the quality of food and the amount of food the same even though it is more expensive now and harder to obtain.” Tier 2    “Absolutely, just the quality that I can buy versus just, "Here's some quesadillas," if I wasn't on it, "Here's some cheese and an egg," and now I can afford to buy much nicer food.” Tier 2    “I felt more comfortable to go and buy different quality of food for the children. I was looking for the prices, to be honest, because even with this [reimbursement rate], serving 12 kids, it's not enough.” Tier 2    “We will have jicama, we will have extra snacks of the stuff that I know that is healthy or I can introduce new foods because I have a little extra income in order to cover that, not just the basic stuff. I can add extra food.” Tier 2    “I think [the higher reimbursement] would allow us, essentially, buying more fresh produce, like fresh vegetables, particularly… it allows us to try more varieties... we have added squash. That's one they like. And also, lettuce.” Tier 1    “It really helped for us to provide more organic fruits and vegetables and also to provide a much larger variety of whole grains, legumes and a lot more organic foods and organic snacks. So really high quality and not having to rely on convenience food or cheaper food or whatever's on sale.” Tier 2    “My variety changed, and I was able to do multiple vegetables during snacks and lunches. Instead of just like when we have apples and peanut butter for snack, I would do apples and peanut butter, and I would do carrots and celery with cream cheese or with ranch dressing that they can dip it into. There was just a little bit, I was able to add a little bit of extra things.” Tier 2    “We were able to shop at farmer's markets, more upscale shopping centers… we were able to get a much wider variety of the fruits and vegetables.” Tier 2    “I was able to offer a second or third fruit or vegetable… To be able to offer watermelon and cantaloupe for lunch, "What would you rather have?" I had that opportunity because there was a little extra.” Tier 2    “That time, I get more money, so I get variety of fruit and vegetables.” Tier 1    “The more I get [in reimbursement], the better quality I can buy for sure.” Tier 1    “It helped them keep buying the food, especially the vegetables and fruits.” Sponsor    “If you had a provider during the pandemic that was receiving that higher reimbursement rate, they would offer more fresh fruits and vegetables, they will offer new fruits as well.” Sponsor  “We have providers that were sending meals home, literally to help families during the pandemic.” Sponsor    “During that time [the pandemic], especially in my tier one areas, I would see providers packing food for their daycare children to take home. These were those that were not being reimbursed on CACFP. These were just additional meals that the provider was packing to make sure that that child had a dinner when that child went home.” Sponsor |
| 13. In-person monitoring waiver |  | | Remote monitoring visits during COVID-19 positively impacted sponsors and providers and supported continued CACFP participation. | “We need it. We were doing this before [in person visits] and it wasn't working and we were like, “Virtual visits work, we see the provider, we see the kids, we see the food, we see everything we need to see, and we didn't have to drive.” It's amazing.” Sponsor    “I don't think there's any way they're going to ever let us go back to just virtual visits, just again because of the integrity of the program, but I think that if they allowed one, it would really help sponsors. It would make a big difference.” Sponsor    “I think the pandemic has made a lot of things very obvious. One is we don't have to be physically everywhere. And I think that will be really good.” Sponsor    “Hybrid would be the best. Only follow up in person for that one unannounced meal, that's fine, and all the other visits can be virtual. On the other hand, if the provider is claiming high numbers for a lot of children and you can't even verify them virtually, then then go see them in person and do it that way. I would think that would be great because it would save on mileage, but then also, they are able to see more meals when they're not traveling.” Sponsor    “One of the beauties that came with a pandemic was being able to not have to do the in person visits. It was very, very good. ... Being that I'm the only Spanish speaking monitor covering [sponsor county name redacted] that means I have to be there for everyone that speaks Spanish in [sponsor county]... it means that I have to go from one spot to the next and my providers are not close by, they're not 15 minutes away from each other... the driving really takes a toll on monitors physically. When you're doing a visit, even if you can keep it very short, the visits never takes 15 minutes... Those visits take anywhere from 30 to 45 to an hour. And it takes five minutes to park five minutes to get out of the car and put all your stuff down, do the visit and another five minutes to go back. Then there's another five minutes to plan where you actually look at the maps and be like, “Can I make it to the next provider?” There's a lot more to having to do in person visits. Where when we used to do them virtually, we were like “Oh, you didn't answer, fine. I'm going to the next one. I'm not going to meet the mealtime because the next one will answer now, I get to see someone's else's meal.” Not like “Oh, it's 12pm and now you are not home, or something happened, and now I can't see anyone's meal.” So that makes it really hard for us.” Sponsor |
| **Perception of Impact Upon Return to Tiers** |  | |  |  |
| 14. Lower reimbursements inadequate | a. Increased costs to parents | | Providers will increase childcare rates and serve fewer meals/snacks or ask parents to bring meals with a lower CACFP reimbursement. | “I have to kind of crunch the numbers and see if I might have to go more expensive, I may have to charge more if the food program is kind of not helping me fulfill the food cost and what it's worth for me to serve food.” Tier 2  “I'm going to eventually have to pass it on to the parents, if they want me to still keep providing all the food.” Tier 2    “I'm probably going to have to increase my rates more significantly to cover that amount… instead of doing a 3% raised rate, increase in tuition, we may have to go up to 10% to help offset the cost of the food. Tier 2  “I may reevaluate what I'm going to do because being on tier two really helps but if I'm not able to be on the tier one, I may have to go back to having parents bring food or I provide partial amounts of food and the parents a partial. We're just going to have to see how it goes.” Tier 2    “I don't plan on closing my family childcare, but it may come to me no longer being able to provide food for the families in my program.” Tier 2    “You see them pulling back a little bit so they're not offering a second serve enough. The child says, “Hey, can I have more food?”, and we see them say, “Oh, no, you've had enough.” Now you see them kind of pulling back a little bit being a little more conservative and a little more reserved on the amounts that they're offering. For instance, they're not willing to offer more than the required amounts now.” Sponsor |
|  | b. Leave CACFP | | Providers may leave CACFP with a lower reimbursement. | “It may impede our ability to participate in the food program if we see a continued reduction in checks.” Tier 2    “Yes, definitely [the lower reimbursement will impact CACFP participation]. Sometimes when I have a new enrollment, I'm not even updating the information to get the reimbursement. It's not even motivating me to spend that much time… for $2, I'm not losing much. It's not so motivating to participate.” Tier 2    “… if the provider is not getting enough, they would not even be part of it. So yes, I think it [lower reimbursement] will [impact CACFP participation by FCCH]. Because it's not going to be enough for them to follow rules.” Tier 1    “I think they [other providers] would be less likely to enroll or stay on the program because of the high cost of food. It's cheaper to have families provide food themselves.” Tier 2    “The lower tier is going to give me less money. But on the other hand, food prices have risen times three. So, it's not just the lower tiers, it's the food pricing in general that's going to affect what I do because I still have to feed the kids, and I still have to feed them really well. So, I don't foresee that I'm going to be able to stay open actually past next May.” Tier 2    “It made me question whether I wanted to stay in the program, because the amount has had gone down, noticeably. And I still had to feed them the same food. I felt like it was costing me money once the reimbursement rate went down.” Tier 1    “A lot of our providers are going to start dropping down to tier two… will we start losing more providers because they don't want to do the work and it's not worth the money? They feel like at that tier two rate, it's not worth it to do the work.” Sponsor    “We ended up losing two of our tier two providers… we lost them once the tier reimbursement rate went back to the tier one, tier two. So, I think that that in itself just kind of showed how much it can benefit to have the higher reimbursement rate.” Sponsor    “We did have a lot of providers who left the program after that COVID reimbursement was removed. Unfortunately, we did lose some providers. Prior to COVID they were at a tier two reimbursement, and they were okay with that and I think they got a glimpse of what a tier one reimbursement rate would be. So then going back to tier two, it really discouraged them, and they saw the big difference in what they were going to now make. I think a lot of them kind of took it as that's not very fair and like almost as if we were picking on them.” Sponsor    “We were afraid of once the pandemic was ending and the tier one for all was ending, that we were going to lose a lot of providers, which we did. We lost a lot of providers that were tier two because they're like, “It's not worth it, having to do all of this, serving this where I could be serving whatever I want, whatever the parents want, and I'll have to fight them over the milk just to get a couple cents.” Sponsor |
|  | c. Reduce food quantity, quality, & variety | | Providers will reduce the quantity, quality and variety of foods with a lower CACFP reimbursement. | “I'm thinking of other ways to cut down my expenses. Either food or my staff, somebody has to compromise to reduce the expenses or additional expenses that I'm incurring.” Tier 2    “We will limit our variety a bit… we would do kiwi and fresh fruit salads and mangoes and try different things that kids at home may not get a chance to try. And now with a lower [reimbursement], I almost want to go... "Hey, apple is a fruit, it is a good fruit, and they eat it, and they'll like it, and they won't waste it." So, you don't want to waste as much money in case they don't like something new….” Tier 1  “Yeah, it is already affecting [it]. I am looking for the ways to cut down the cost. That's a direct effect on my quality. I reduced the number of times I provide a snack.” Tier 2    “It does limit the variety and some of the more expensive meals to create for the children… you stick with the standards of a whole grain grilled cheese sandwich… and you tend to look for the cheaper stuff. And a lot of it is unhealthy but less expensive as opposed to giving them a bigger variety.” Tier 1    “They'll get the $2 a loaf wheat bread, which they're getting the whole grain but they're not getting a good variety. They're just getting that… they're getting the grain but it's not a good variety. It's one or two things, not like it used to be.” Tier 1  “I think it's going to make it so that we aren't able to provide as much of a variety and that we're also going to have to provide more bulk foods. The children, they're going to have to eat the same thing in the menu plan twice a week, three times a week, versus having something different for every meal. The variety really helps prime their palate for being an adult and giving them that opportunity is really important.” Tier 2    “Now that they've gone back to the lower you see more of like canned peaches, more of the can mandarins opposed to the fresh fruits and vegetables.” Sponsor    “I was talking with some providers and they're like, “Well, I can’t now look at organic stuff or berries and certain items that I like to buy for the kids. I guess I'm gonna be buying apples and oranges, you know, because that's kind of all that I'm gonna be able to afford because I can't put any more out of my pocket.” Providers already put a lot of out of their pocket in order to cover the meals that go into the daycare… I believe that the quality of food has downgraded. I think the kids are getting much more what the providers can find on sale… I heard one provider say, “Well, I guess they're back on oranges and apples because I can't afford blueberries and cantaloupe and watermelon.” Sponsor |
