# Supplementary material for: Provider, sponsor and family perceptions of Child and Adult Care Food Program (CACFP) participation and COVID-19 reimbursement increases
Source: Public Health Nutr. 2025 Nov 3;28(1):e195. doi: 10.1017/S1368980025101389 (PMC12722094; doi:10.1017/S1368980025101389)
Supplement: Bacon et al. supplementary material 2 — Bacon et al. supplementary material [file S1368980025101389sup002.docx]

Supplemental B. Coding Scheme

**PROVIDERS AND SPONSORS**

Benefits

- Reimbursements
- Support families with food security
- Healthy meals and snacks
- Nutrition education
- Parental perception

Challenges

- Paperwork/daily report
- Inadequate reimbursement
- Meal Patterns/Nutrition Standards
- Monitoring visits
- Serious Deficiency process
- Food Waste
- COVID challenges – child enrollment, finding eligible foods, decreased sponsor admin funds
- Inflation – negative impact on budget, pay out of pocket, decreased variety/quality/quantity, labor costs

Reasons for leaving

- Return to tiers
- Inadequate reimbursement levels
- CACFP regulations, daily reporting, inspections
- Decreased child enrollment
- Professional changes (retirement)

Facilitators

- Elimination of tiers – encouraged CACFP participation, increased food purchases/balanced out inflation, improved quality and variety foods, easier to shop and pay for additional staff time, supported families with food insecurity
- Elimination of in-person monitoring visits

Perception of impact upon return to tiers

- Inadequate reimbursement
- Pass food costs along to parents, stop serving meals/snacks
- Increasing childcare rates
- Paying out of pocket
- Leave CACFP
- Reduce variety, quality, quantity

**FAMILY INTERVIEWS**

Importance of meals/snacks offered by childcare

Awareness of CACFP

Awareness and perception of quality and quantity of meals/snacks served

Perception of changes in childcare during pandemic

| **I. General Perceptions of CACFP**  These themes and subthemes reflect the perceptions of providers, sponsors and families on the benefits and challenges of CACFP in general, meaning they are not necessarily specific to the removal of tiers and increased CACFP reimbursements. | | |
| --- | --- | --- |
| **Theme** | **Subtheme** | **Description** |
| **Benefits of and Reasons For CACFP Participation** |  |  |
| 1. Reimbursements |  | CACFP reimbursements offset food costs and help providers buy higher quality food. |
| 2. Consistent meals/snacks and food security |  | Providing kids with consistent and healthy meals/snacks, supporting children who may experience food insecurity. |
| 3. Nutrition standards, education, and resources |  | Increases providers’ awareness about what and how to feed children through nutrition standards, education and resources. |
| **Challenges of CACFP Participation** |  |  |
| 4. Regulations | a. Burdensome daily reporting | CACFP paperwork and daily reporting is burdensome and can be technologically challenging. |
|  | b. Tier 2 requires more paperwork and sponsor support | It is administratively burdensome for sponsors to support Tier 2 providers with tiering determination; provider also struggle with the meal benefit forms. |
|  | c. Nutrition standards | Meeting the CACFP nutrition standards and meal pattern requirements is difficult or there is difficulty finding CACFP eligible foods. |
|  | d. Monitoring visits | In-person monitoring visits are challenging. |
|  | e. Serious deficiencies | Serious deficiency hinders CACFP participation, is perceived as being “too harsh”, time-consuming for sponsors, or creates language inequities. |
| 5. Inadequate reimbursement | a. Tiered rates are inadequate and unfair | Tier 1 and 2 reimbursement rates are inadequate to cover food costs and not worth the effort. The tiered system is disliked and perceived of as unfair; providers recommend a universal tier. |
|  | b. Inadequate sponsor administrative funds | Sponsors perceive the CACFP administrative funds are inadequate. |
| 6. Food waste |  | Children do not eat or drink all the food and beverages, resulting in food waste. |
| **Family Perceptions of CACFP and Child Care Meals** |  |  |
| 7. Awareness of and appreciation for CACFP |  | Families are aware their provider participates in CACFP and may express gratitude for the program. |
| 8. Importance of meals/snacks offered with childcare | a. Families value childcare meals/snacks | Families indicate healthy, high quality, and a variety of meals and snacks are provided to their children and that this is important to them and may be a consideration for selecting a childcare provider. Providers and sponsors also indicate CACFP has a positive parental perception. |
|  | b. Negative impacts anticipated if provider stops offering food | If providers stopped offering meals and snacks as part of their care, this would results in challenges to the families and may result in them changing childcare providers. |
| **II. Perceptions of CACFP During COVID-19 Waiver Period (Higher Reimbursement)**  These themes and subthemes reflect perceptions of CACFP when the tiers were waived, and the impact of the temporary higher reimbursement and subsequent reinstatement of tiers from interviews with providers and sponsors. | | |
| **Theme** | **Subtheme** | **Description** |
| **COVID-19 and Inflation Challenges** |  |  |
| 9. Difficulty finding CACFP-eligible food |  | Pandemic supply chain issues and inflation impacted providers’ ability to find CACFP-eligible foods. |
| 10. Negative impacts on food and business budget |  | Providers indicated negative impacts to their business and food budgets due to COVID-19 and inflation. Sponsors observed providers choosing cheaper, frozen, or packaged food items due to budget constraints. |
| 11. Decreased child enrollment |  | Child enrollment at family childcare homes decreased due to COVID-19, leading to decreased CACFP meal/snack claiming |
| **Facilitators to CACFP Participation** **During COVID-19** |  |  |
| 7. Removal of tiered reimbursements | a. Supported enrollment and retention | Encouraged new and existing providers to join or stay on CACFP. |
|  | b. Offset inflation | Higher reimbursements balanced out rising food costs due to inflation. |
|  | c. Better meals/snacks | The higher reimbursement resulted in improved quality and variety and increased the quantity of meals/snacks served, and made it easier for providers to shop for food. |
| 8. In-person monitoring waiver |  | Remote monitoring visits during COVID-19 positively impacted sponsors and providers and supported continued CACFP participation. |
| **Perception of Impact Upon Return to Tiers** |  |  |
| 9. Lower reimbursements inadequate | a. Increased costs to parents | Providers will increase childcare rates and serve fewer meals/snacks or ask parents to bring meals with a lower CACFP reimbursement. |
|  | b. Leave CACFP | Providers mat leave CACFP with a lower reimbursement. |
|  | c. Reduce food quantity, quality, & variety | Providers will reduce the quantity, quality and variety of foods with a lower CACFP reimbursement. |
