# Supplementary material for: Provider, sponsor and family perceptions of Child and Adult Care Food Program (CACFP) participation and COVID-19 reimbursement increases
Source: Public Health Nutr. 2025 Nov 3;28(1):e195. doi: 10.1017/S1368980025101389 (PMC12722094; doi:10.1017/S1368980025101389)
Supplement: Bacon et al. supplementary material 3 — Bacon et al. supplementary material [file S1368980025101389sup003.docx]

Supplemental C. FCCH Provider, Sponsor, and Family Interview Guides

**Provider Interview Guide**

*[Domain: CACFP Participation]* 5 Questions

1. (Ice breaker) It looks like you said in your survey that you’ve been participating in the Food Program for XX years, is that right?
2. Are you part of the Early Head Start Child Care Partnership program?
   1. If unclear what that is – are you required to participate in the Food Program?
3. Why are you participating in the Food Program?
4. What are the greatest benefits to participating in the Food Program?
5. What are the greatest challenges to participating in the Food Program?

*[Domain: Perception of CACFP Tiers]* 3 Questions

1. What do you think about the Food Program’s tiered reimbursement rates? (If participant is unclear what tiered reimbursement rates are, please read this sentence: Tiered reimbursement rates refer to the amount of money a child care provider receives for serving up two meals and one snack to the children in their care. Rates are determined by certain criteria, and there are two Tiers – Tier 1 reimbursement rates are the highest, Tier 2 reimbursement rates are the lowest.)

*[To be read to Tier 1 interviewees]*

Prior to COVID-19, you could have received **$4.78 per child if you claimed breakfast, lunch, and a snack.** During COVID-19, you could have received **$5.67 per child if you claim breakfast, lunch, and a snack.**

*[To be read to Tier 2 interviewees]*

Prior to COVID-19, you could have received **$2.29 per child if you claimed breakfast, lunch, and a snack**. During COVID-19, you could have received **$5.67 per child per day if you claim breakfast, lunch, and a snack.**

1. Did the higher COVID-19 reimbursement rate affect participation in the Food Program for you or other family child care home providers?
   1. Probe: In what ways?
   2. Are they more likely to enroll or stay on the program or does this not have an impact?
2. In June of 2023, the higher COVID-19 rates came to an end and Tier 1 and Tier 2 rates returned. Do you think this return to Tier 1 and Tier 2 rates will impact Food program participation by family child care homes?
   1. Probe: Are they less likely to enroll or stay on the program or does this not have an impact?

*[Domain: Financial Viability of FCCH]* 2 Questions

Let’s talk some more about the higher COVID-19 Food Program reimbursement rate and how it may have impacted you and your family child care home.

1. How did the higher reimbursement rate impact the overall food budget for your family child care home?
   1. Probe: How about the number of meals and snacks you can serve at your family child care home?
   2. Probe: How about covering the cost of labor or the amount of time it takes you and/or your staff to plan, prepare, and serve the meals?
2. The higher reimbursement rate came to an end in June 2023. How might this impact your family child care home business?
   1. Probe: How might this affect your ability to keep your business open?
   2. Probe: How might this affect your ability to participate in the Food Program?
   3. Probe: How might this affect your ability to provide meals and/or snacks?

*[Domain: Quality of Meals/Snacks Served]* 5 Questions

*To be read to participant if interviewer deems a review of reimbursement rates is necessary to help the participant answer the questions:* As a reminder, prior to the COVID-19 pandemic, Tier 1 providers received **$4.78** per child for serving breakfast, lunch, and a snack, and Tier 2 providers received **$2.29** per child. During COVID-19, all family child care home providers received a higher reimbursement rate of **$5.67** per child for serving breakfast, lunch, and a snack.

I am going to ask you some questions about food groups you may serve at your family child care home and if these changed with the higher Food Program reimbursement rates.

1. We’ll start with fruits and vegetables *(including fresh, frozen, canned, dried, beans and peas, and vegetables and fruit juice).*
   1. With the higher Food Program reimbursement rates:
      1. Has the variety, or number of different types, changed? (Different types of vegetables include dark green, red, and orange, beans and peas (legumes), starchy vegetables, and others.)
      2. Has the quality changed?
      3. Has the amount you serve changed?
      4. Has there been any impact on the amount of seasonal or locally grown produce you serve?
2. The next food group is grains (*including enriched and whole grains, and breakfast cereals.)*
   - 1. Has the variety, or number of different types, changed?
     2. Has the quality changed?
     3. Has the amount you serve changed?
3. Meat and meat alternatives (*including meat, poultry, fish, eggs, nuts and seeds, tofu and other soy products, and nut butters.)*
   - - 1. Has the variety, or number of different types, changed?
       2. Has the quality changed?
       3. Has the amount you serve changed?
4. For Dairy, *(including milk, yogurt, and cheese.)*
   - - 1. Has the variety, or number of different types, changed?
       2. Has the quality changed?
       3. Has the amount you serve changed?
5. Do you think the higher reimbursement rate is enough for family child care home providers like yourself to purchase foods that meet Food Program requirements?
   1. Probe: Has this changed compared to when the pre-COVID-19 reimbursement rates were in place?

*[Domain: Perceived Food Security of Children/Families Served]* 2 Questions

1. How do you think your participation in the Food Program impacts families in your care that may not have enough food at home, if at all?
2. How do you think the higher COVID-19 reimbursement has impacted families in your care that may not have enough food at home, if at all?

*[Domain: Impact of Inflation on Provider & Food Program]* 2 Questions

1. Have the rising costs of food, gas, and other goods and services due to inflation impacted your family child care home business? If yes, how so?
   1. Probe: Has this impacted your ability to purchase foods that meet the Food Program requirements?
   2. Probe: Has this impacted the cost of labor or the amount of time it takes you and/or your staff to plan, prepare, and serve the meals?
2. Is there anything else that you would like to share with us about the reimbursement rates for the Food Program?

*[Domain: Conclusion]*

In appreciation of your time, we would like to send you a gift card for $50.00.

**Sponsor Interview Guide**

*[Domain: CACFP Participation]* 3 Questions

1. What are some benefits for family child care homes that participate in CACFP?
2. What barriers do family child care homes experience participating in the Food Program?
3. Why have family child care homes that were previously enrolled in the Food Program dropped off?

*[Domain: Perception of CACFP Tiers]* 5 Questions

1. What are your overall thoughts about the Food Program’s tiered reimbursements?
2. How do you think Food Program reimbursement tiers impact program participation for family child care home providers?
   1. Probe: Are there differences in Food Program participation between Tier 1 and Tier 2 providers?
3. What are some of the main differences in sponsoring Tier 1 and Tier 2 providers?
   1. Probe: Are there unique challenges in sponsoring Tier 1 providers? Tier 2 providers?

Prior to the COVID-19 pandemic, Tier 1 providers received **$4.78** per child for serving breakfast, lunch, and a snack, and Tier 2 providers received **$2.29** per child. During the pandemic, all child care home providers could have received a single reimbursement rate of **$5.67** per child for serving breakfast, lunch, and a snack. This single reimbursement rate refers to the higher COVID-19 Tier 1 rate that all providers received – I’ll be referring to it as the ‘single reimbursement rate’ for the remainder of the interview.

1. How did the single reimbursement rate impact family child care home Food Program participation at your sponsoring organization, if at all?
   1. Probe: Were there differences in Food Program participation between Tier 1 and Tier 2 providers pre-pandemic? How about during the pandemic?
   2. Probe: Do you think there have been participation changes at other sponsoring organizations?
2. In June of 2023, the single reimbursement rate came to an end and tiering was reinstated. How do you think this reinstatement of tiering will impact Food program participation for family child care homes?
   1. Probe: Do you think there will be differences in Food Program participation between Tier 1 and Tier 2 providers?

*[Domain: Quality of Meals/Snacks Served]* 2 Questions

1. How did the single reimbursement rate affect the meals and snacks served at the child care homes your organization sponsors, if at all?
   1. Probe: How about the types or variety of foods served for meals and snacks?
   2. Probe: How about the quality of foods served?
   3. Probe: How about the amounts served?
2. Do you think the single reimbursement rate was enough for family child care home providers to purchase foods that meet CACFP requirements?
   1. Probe: Has this changed compared to tiered reimbursement rates prior to COVID-19?

*[Domain: Financial Viability of FCCH and Sponsors]* 3 Questions

1. What challenges exists for sponsors with a large number of FCCHs? A small number of FCCHs?
   1. Probe: What challenges, if any, do sponsors encounter in supporting FCCHs as FCCH CACFP enrollment continues to decrease?
   2. Probe: Are there any challenges related to traveling for your sponsoring organization?
      1. Probe: Any unique challenges related to urban travel? Rural travel?
2. Do you think the return to tiering will impact Food Program claims made by family child care home providers?
   1. Probe: How might this affect family child care home sponsor administrative funds?
   2. Probe: How about rising costs of food, gas, and other goods due to inflation – how might affect Food Program claims by providers now that tiers are reinstated?
3. Does your sponsoring organization have the resources or capacity to communicate effectively with providers whose first language is not English?

*[Domain: Perceived Food Security of FCCH Providers and Children Served]* 4 Questions

1. Do you know if any family child care providers you serve experience food insecurity, meaning they don’t have access to enough food for themselves or their family?
   1. Probe: Do you assess hunger experienced by the family child care home providers you serve.
   2. Probe if assessed: Do you address hunger of the family child care home providers you serve. If so, how?
   3. Probe: Does food insecurity differ between Tier I and Tier II providers?
2. Did the single reimbursement rate impact food security for the family child care home providers you serve?
   1. Probe: Did this differ between Tier I and Tier II providers?
3. Do you know if the single reimbursement rates have impacted food security of the families served by family child care homes? If so, how?
4. Is there anything else that you would like to share with us about the reimbursement rates for the Food Program?

*[Domain: Conclusion]*

In appreciation of your time, we would like to send you a gift card for $50.00.

**Family Interview Guide**

*[Domain: Ice breaker]* 2 questions

1. How many of your children attend your current family child care home?
2. How long has/have your child/ren been attending your current family child care home?

*[Domain: CACFP Participation]*  4 Questions

1. Are free meals and snacks provided by your family child care provider. If yes, about how many meals and snacks are served per day?
2. When looking for a family child care provider for your child/ren, do you consider whether they provide free meals? Why or why not?
   1. Probe: How important is it that your family child care home provider serves meals and snacks?
3. Do you know if your family child care participates in the Food Program?
   1. Yes
   2. No *(skip Q4)*
4. What do you know about the Food Program?
   1. Probe: What are your thoughts or opinions on the Food Program?
   2. Probe: Providers on the Food Program only get a partial reimbursement for the food they serve to children in their care. What are your thoughts on this partial reimbursement?

*[Domain: Perceived Financial Viability of FCCH Business]* 2 Questions

1. In the past year, has the cost of child care changed at the family child care home your child/ren attend? If so, why do you think it has or hasn’t changed?
2. In the past year, have there been any changes to the number of meals and/or snacks provided to your child/ren at the family child care home they attend? If so, why do you think there has been a change?

*[Domain: Perceived Quality of meals/snacks served]* 3 Questions

1. What do you know about the meals and snacks your family child care provider serves?
   1. Probe: How do you know what your child/ren will be eating for meals and snacks?
      1. Probe: Are you provided with the menu? Does your family child care provider post the menu?
2. How important is it to you that your child/ren are served healthy meals and snacks?
   1. Probe: How about the variety, or number of different types of foods served for meals and snacks?
   2. Probe: How about the quality of the meals and snacks served? Quality meaning fresher and less processed foods.
      1. Not important
      2. A little bit important
      3. Important
      4. Very important
3. How do you feel about the amount of food served for meals and snacks at your family child care home?
   1. Probe: Do(es) your child/ren often feel hungry after coming home from child care?

*[Domain: Perceived Food Program Impacts on Providers and Families]* 3 Questions

1. Has the provider at the family child care home your child/ren attend ever asked you to provide meals and snacks for your child/ren because they can no longer provide them? If yes, did they say why?
   1. Probe (if yes): If yes, how has this impacted you and your family, if at all?
   2. Probe (if no): If they were to stop offering meals/snacks and ask you to them for your child, how would this impact you and your family, if at all?
2. Is there anything else that you would like to share with me about the meals and snacks provided at the child care home your child(ren) attend(s)?

*[Domain: Conclusion]*

In appreciation of your time, we would like to send you a gift card for $50.00.
